# RNA sequencing of db/db mice liver identifies lncRNA H19 as a key regulator of gluconeogenesis and hepatic glucose output

**DOI:** 10.1038/s41598-017-08281-7

**Published:** 2017-08-16

**Authors:** Neha Goyal, Ambily Sivadas, K. V. Shamsudheen, Rijith Jayarajan, Ankit Verma, Sridhar Sivasubbu, Vinod Scaria, Malabika Datta

**Affiliations:** 1 CSIR-Institute of Genomics and Integrative Biology, Mall Road, Delhi, 110007 India; 2 Genomics and Molecular Medicine, CSIR-Institute of Genomics and Integrative Biology, Mathura Road, Delhi, 110025 India; 3 GN Ramachandran Knowledge Centre for Genome Informatics, CSIR-Institute of Genomics and Integrative Biology, Mathura Road, Delhi, 110025 India; 4 Academy of Scientific and Innovative Research, Training and Development Complex, CSIR Campus, CSIR Road, Taramani, Chennai, India

## Abstract

Liver plays a key role in maintaining glucose homeostasis and impaired hepatic glucose metabolism is associated with type 2 diabetes. In the present study, we used RNA sequencing to profile the transcriptome of the livers of diabetic db/db mice as compared to the normal db/+ mice and identified 218 differentially expressed genes. Amongst these, there were 3 lncRNAs that were significantly downregulated and H19 was the most altered lncRNA in the livers of db/db mice. H19 expression significantly correlated with the expression of genes of the glycolysis and gluconeogenesis pathways, which suggest that altered hepatic H19 levels can directly or indirectly modulate their expression. Inhibition of H19 using specific siRNA in HepG2 cells and primary mouse hepatocytes significantly increased the levels of gluconeogenic genes. This was subsequently accompanied by increased hepatic glucose output. Further,H19 depletion in HepG2 cells impaired insulin signaling and increased nuclear localization of FoxO1, an important transcriptional regulator of gluconeogenic gene expression. Our results reveal a novel link between decreased H19 levels and impaired gluconeogenesis via regulation of FoxO1 nuclear levels. These put forth interesting observations on the regulatory role of H19 in altering hepatic physiology during diabetes.

## Introduction

The recent transcriptome annotation of the human genome^1^ and of the genomes of vertebrate model organisms including mouse^2^, rat^3^ and zebrafish^4^ has uncovered a very large number of transcripts which do not have the potential to encode for proteins. Long noncoding RNAs (lncRNAs) form a major proportion of these novel transcripts and a comprehensive list of such lncRNAs can be obtained from GENCODE^1^. Its latest transcript annotation annotated at least 15,787 human lncRNA genes representing  ~27,720 (v 2.6) transcripts and 11,017 genes representing  ~15,300 (v M13) transcripts in mouse. By definition, lncRNAs encompass transcripts longer than 200 nucleotides without any coding potential. They are mostly transcribed by the RNA polymerase II complex and often are 5′-capped, spliced and polyadenylated^5^. LncRNAs can be expressed from intergenic regions of the genome (lincRNA) or can be transcribed in the antisense direction to the protein coding genes or can also be derived from the introns of the protein coding genes (intronic lncRNAs)^1^. LncRNAs exhibit tissue specific expression and are generally less conserved than coding transcripts. In the recent past, they have gained importance as major regulatory molecules in health and disease. A number of reports have shown the role of lncRNAs in development, differentiation, cancer metastasis and in metabolic disorders^6, 7^, yet the physiological significance of most lncRNAs at the organism level, as a whole, is largely unknown.

Type 2 diabetes is a complex metabolic disorder characterized by hepatic insulin resistance and deregulated glucose metabolism^8^. A major hallmark of this hepatic anomaly is increased hepatic glucose production due to impaired gluconeogenesis or glycogenolysis^9, 10^. Gene expression profiles in rodent models of type 2 diabetes revealed increased expression of hepatic gluconeogenic genes^11–13^ and human subjects with mild and severe type 2 diabetes have elevated fasting and postprandial glucose levels^14^. Pre-diabetic subjects also have similar metabolic abnormalities resulting in elevated fasting glucose levels associated with impaired insulin induced suppression of gluconeogenesis. These suggest that alterations in these pathways set in quite early during the development of type 2 diabetes^15^.

The implications and roles of lncRNAs in diabetes is not completely understood. A recent study uncovered lncRNAs that are tissue specific, dynamically regulated and abnormally expressed in pancreatic β-cells of type 2 diabetes patients suggesting their potential roles in β-cell function^16^. Certain lncRNAs have also been shown to map to established type 2 diabetes associated loci^17, 18^. A recent report has demonstrated abnormal H19 lncRNA levels in the skeletal muscle during diabetes and its role in impairing insulin signaling and decreasing glucose uptake^19^. The liver enriched lncRNA, lncLSTR regulates systemic lipid homeostasis by regulating the TDP-43/FXR/apoC2-pathway^20^ and upregulation of lncRNA MEG3 in the liver of HFD and ob/ob mice leads to elevated hepatic insulin resistance via FOXO1 over expression^21^. MEG3 downregulation also affects insulin synthesis and secretion in mouse pancreatic β-cells and it’s *in vivo* knockdown leads to impaired glucose tolerance^22^. A fasting induced lncRNA, liver glucokinase repressor (lncLGR) suppresses glucokinase activity and regulates glycogen storage in the mouse liver^23^. In spite of these few reports of the roles of lncRNAs in the liver, their detailed roles and the metabolic pathways that they might regulate is still an unexplored territory. Since deregulated glucose metabolism is associated with type 2 diabetes, bridging these two metabolic domains could significantly contribute towards understanding the disease pathology and may open up new resources for therapeutic interventions.

In the present study, we sought to explore differentially altered lncRNAs in the livers of diabetic db/db mice, assess their physiological effects and correlate them to the altered hepatic physiology during diabetes.

## Results

### RNA Sequence Data Generation and Reference Alignment

Sequencing was performed on RNA isolated from livers of biological replicate samples of 10–12 week old male normal (C57BLKs-db/+) mice weighing 22.05 ± 1.59 g with blood glucose levels: 151 ± 27 mg/dl and diabetic (C57BLKs-db/db) mice weighing 46.3 ± 8 g with blood glucose levels: 463 ± 98 mg/dl, respectively. Transcriptome sequencing was performed using strand-specific cDNA libraries prepared from ribosomal RNA-depleted total liver RNA. We generated a total of approximately  ~70 million and  ~74 million 101 bp-long paired-end raw sequencing reads from the normal and diabetic samples, respectively (Supplementary Table S1). After removing adapter contamination and trimming low quality bases, the filtered reads were mapped to the mouse reference genome (GRCm38/mm10) as detailed in the “Methods” section and processed for downstream analysis.

### Differential Expression Analysis of Liver Transcriptomes

**Figure 1:**
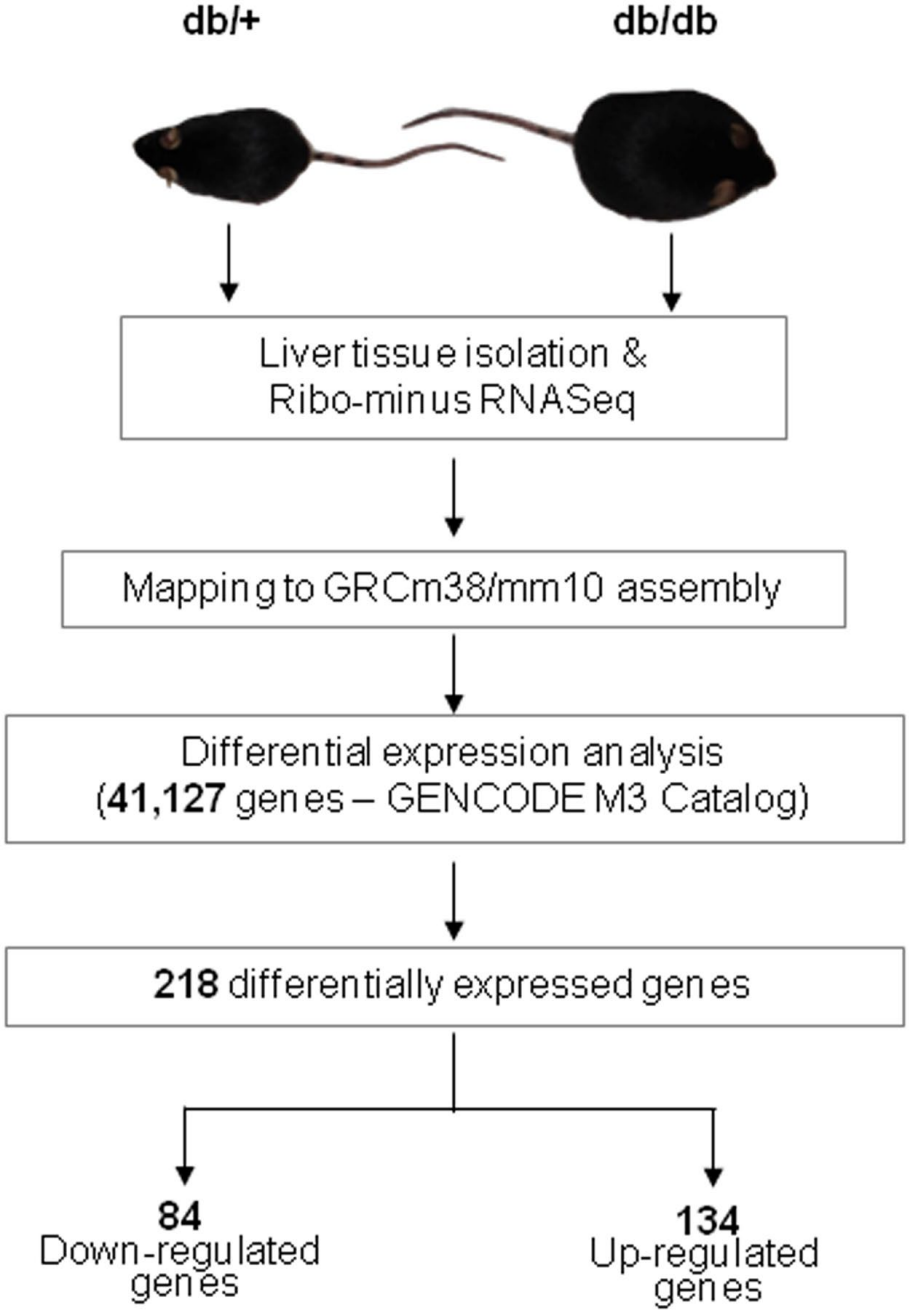
Schema of the experimental workflow. Summary and overview of experimental and computational workflow adopted in this study to identify genes differentially expressed between normal (db/+) and diabetic (db/db) mice liver.

We used the GENCODE (M3) Catalog gene and transcript annotations to evaluate differentially expressed genes. The GENCODE (M3) catalog is annotated for 41,127 mouse genes. The expression of genes was quantified as FPKM (Fragments Per Kilobase of transcript per Million mapped reads) values. Cuffdiff was used to normalize the FPKM values and to evaluate differential gene expression between diabetic and normal mice liver transcriptomes comprising of two biological replicates each. Figure 1 summarizes the steps undertaken and provides an overview of the data analysis workflow.

**Figure 2:**
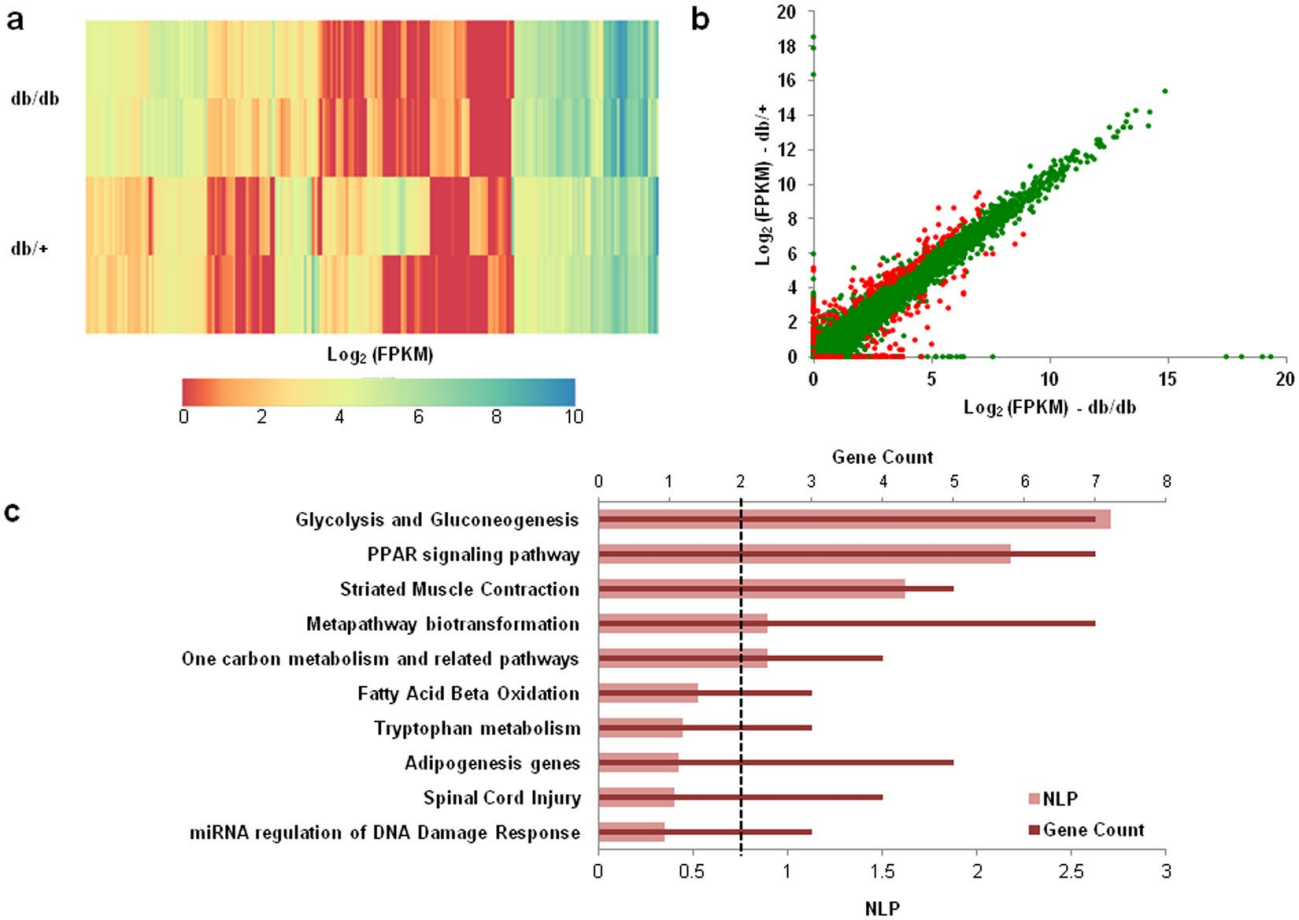
Representation of differentially expressed genes in the db/db mice liver. (**a**) Heatmap representing the expression pattern of 218 differentially expressed genes (as obtained from RNA sequencing) in the livers of diabetic (db/db) mice as compared to those of normal (db/+) mice. (**b**) Scatter plot comparing the mean expression profiles of all genes with FPKM >  = 1 in at least one sample within normal and diabetic mice liver. The significantly differentially altered genes (218) have been highlighted in red color, while the remaining are indicated in green color. (**c**) Bar plot summarizing the results of pathway enrichment analysis for the differentially expressed (DE) genes. The figure plots NLP (Negative Log10 of the Bonferroni-adjusted P-value) as well as the overlapping gene count for each pathway. The black vertical dotted line denotes a minimum gene overlap count cutoff of 2.

We identified a total of 218 genes including 203 protein coding and 3 lncRNA genes showing significant differential expression between the normal and diabetic samples (Supplementary Table S2). The distribution and expression patterns of the 218 differentially expressed genes in the samples are highlighted in Fig. 2a and b; of these, 134 genes were upregulated and 84 were downregulated.

### Pathway Enrichment Analysis Reveals Pathways Altered In the Livers of db/db Mice

Towards understanding the biological pathways and functions altered in db/db mouse liver, we performed pathway enrichment analysis with the 218 differentially expressed genes using EnrichR (http://amp.pharm.mssm.edu/Enrichr/). Species-specific functional analysis using WikiPathways ontologies revealed that these differentially expressed genes were majorly involved in Glycolysis and Gluconeogenesis and PPAR signaling pathways (Fig. 2c). Both, glycolysis and gluconeogenesis are critical in altering hepatic glucose production during diabetes^9, 24^, although of these, hepatic gluconeogenesis is drastically increased in type 2 diabetes and accounts for most of the increased hepatic glucose production under these conditions^25^. Hepatic Peroxisome proliferator-activated receptor (PPAR) signaling participates in lipid regulation and glucose homeostasis. Activation of PPARγ signaling promotes hepatic lipid accumulation in rodents^26^ and hepatic expression of PPARγ is elevated in rodent models of diabetes and obesity^27^. These suggest that these metabolic pathways, possibly modulated by the differentially expressed genes are critical during the altered hepatic physiology during diabetes.

### LncRNA H19 is Downregulated in the Livers of db/db Mice

**Figure 3:**
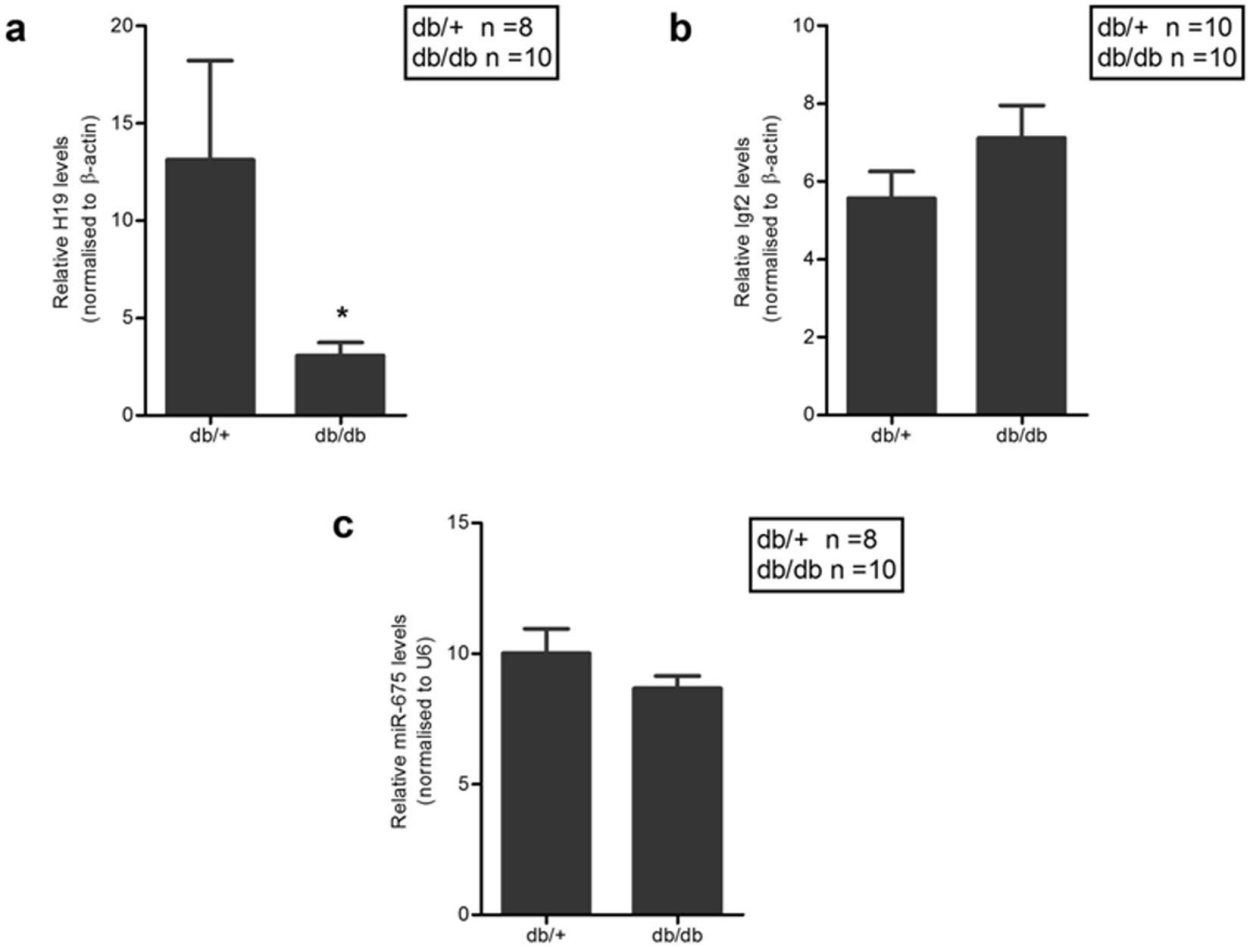
H19 levels are downregulated in the db/db mice liver. (**a**) 1 µg of total RNA was reverse transcribed and expression levels of H19 lncRNA in the livers of db/+ (n = 8) and db/db (n = 10) mice were quantified by qRT-PCR using specific primers. β-actin was used as a normalization control. (**b**) Expression levels of hepatic Igf2 was quantified in db/+ (n = 10) and db/db (n = 10) mice by qRT-PCR using specific primers. (**c**) 1 µg of total RNA was reverse transcribed using specific stem loop primers for miR-675 and its expression was quantified in the livers of db/+ (n = 8) and db/db (n = 10) mice. U6 was used as the normalization control. Data presented are means ± s.e.m. *P < 0.05.

A closer analysis of the three lncRNAs differentially expressed revealed that the lncRNA, H19 was downregulated by  ~7-fold in diabetic mice liver compared to normal mice. The expression levels of H19 was independently validated by quantitative RT-PCR in a larger sample size and as in the RNA sequencing, qRT-PCR also demonstrated that hepatic H19 levels are significantly downregulated in the db/db mouse liver (Fig. 3a).

H19 is a 2.3-kb long noncoding RNA that is highly expressed during embryogenesis but is strongly down regulated after birth^28^. It is transcribed from a conserved imprinted gene cluster on chromosome 7 in mouse and on chromosome 11 in human just adjacent to the Igf2 locus. These two genes are reciprocally imprinted and while H19 is maternally expressed, Igf2 is expressed from paternal allele^29^. Since H19 was down regulated in diabetic mice, we, therefore, sought to assess hepatic Igf2 levels. As shown in Fig. 3b, there was no significant change in Igf2 transcript levels in the db/db mice liver as compared to normal mice liver. In addition, a microRNA, miR-675 is embedded in the first exon of H19 and is reported to be co-expressed with H19^30^. It functions to regulate placental growth, maintains adult hematopoietic stem cells^31^ and also promotes skeletal muscle differentiation and regeneration^32^. However, in this case, hepatic levels of miR-675 did not show any significant alteration in the db/db mice (Fig. 3c) indicating that this microRNA and its host (H19) are possibly not coregulated, at least, in the diabetic mice liver.

H19 levels are also decreased in skeletal muscles of type 2 diabetic patients and HFD fed mice^19^ and our data, additionally, prompted us to hypothesize that H19 could be an important contributor during the pathogenesis of type 2 diabetes by also affecting hepatic metabolic pathways.

### LncRNA H19 expression significantly Correlates with Glycolytic and Gluconeogenic genes

To gain a better understanding of the functional significance of the downregulation of H19 expression in diabetic mice liver, we performed an expression correlation analysis on the RNAseq dataset to identify genes, which are significantly correlated with the expression levels of H19. From a list of 41,127 genes expressed in the liver, we identified a total of 2,284 genes whose expression significantly correlated with H19 (Pearson correlation coefficient (absolute value) of >0.95 and p < 0.05).

**Figure 4:**
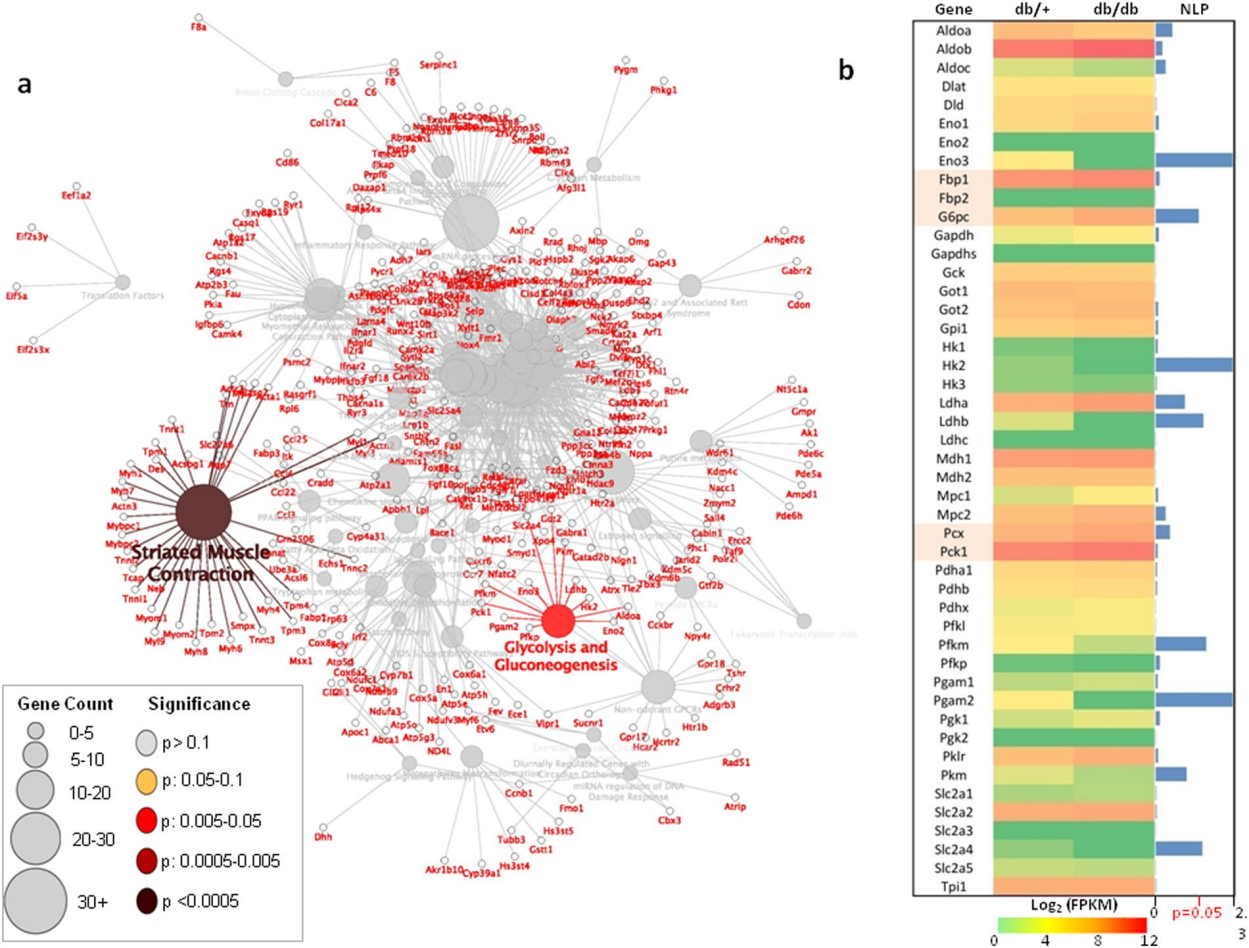
Gluconeogenic genes are differentially expressed in the diabetic mice liver and correlate with H19 expression levels. (**a**) Network representation of the biological pathways enriched among 2284 genes that correlated with H19 expression. (**b**) Mean expression pattern of all glycolysis and gluconeogenesis genes between diabetic (db/db) and normal (db/+ ) mice livers as obtained from the RNA sequencing analysis. The bars in blue depict NLP (negative logarithm (log10) of the p-value) of differential expression. Highlighted gene names indicate genes specific to the gluconeogenic pathway.

Further pathway annotation network analysis of these correlated genes using ClueGO demonstrated significant enrichment of genes (p < 0.05) involved in two major pathways, striated muscle contraction and glycolysis and gluconeogenesis (Fig. 4a). Since glycolysis and gluconeogenesis is of significance in hepatic metabolism (as described above), a detailed inspection of all genes involved in the glycolysis and gluconeogenesis pathways revealed significant (p < 0.05) differential expression of at least 7 major genes in db/db mice liver including G6pc (glucose-6-phosphatase), one of the major players of the gluconeogenic pathway (Fig. 4b). RNA sequencing revealed other gluconeogenic genes mainly Pck1 (phosphoenolpyruvate carboxykinase 1), Pcx (pyruvate carboxylase) and Fbp1 (fructose bisphosphatase 1) to be upregulated in the diabetic mouse (db/db) liver, although with a very modest fold change (Fig. 4b).

### Regulation of Gluconeogenic Genes’ expression by H19 inhibition

**Figure 5:**
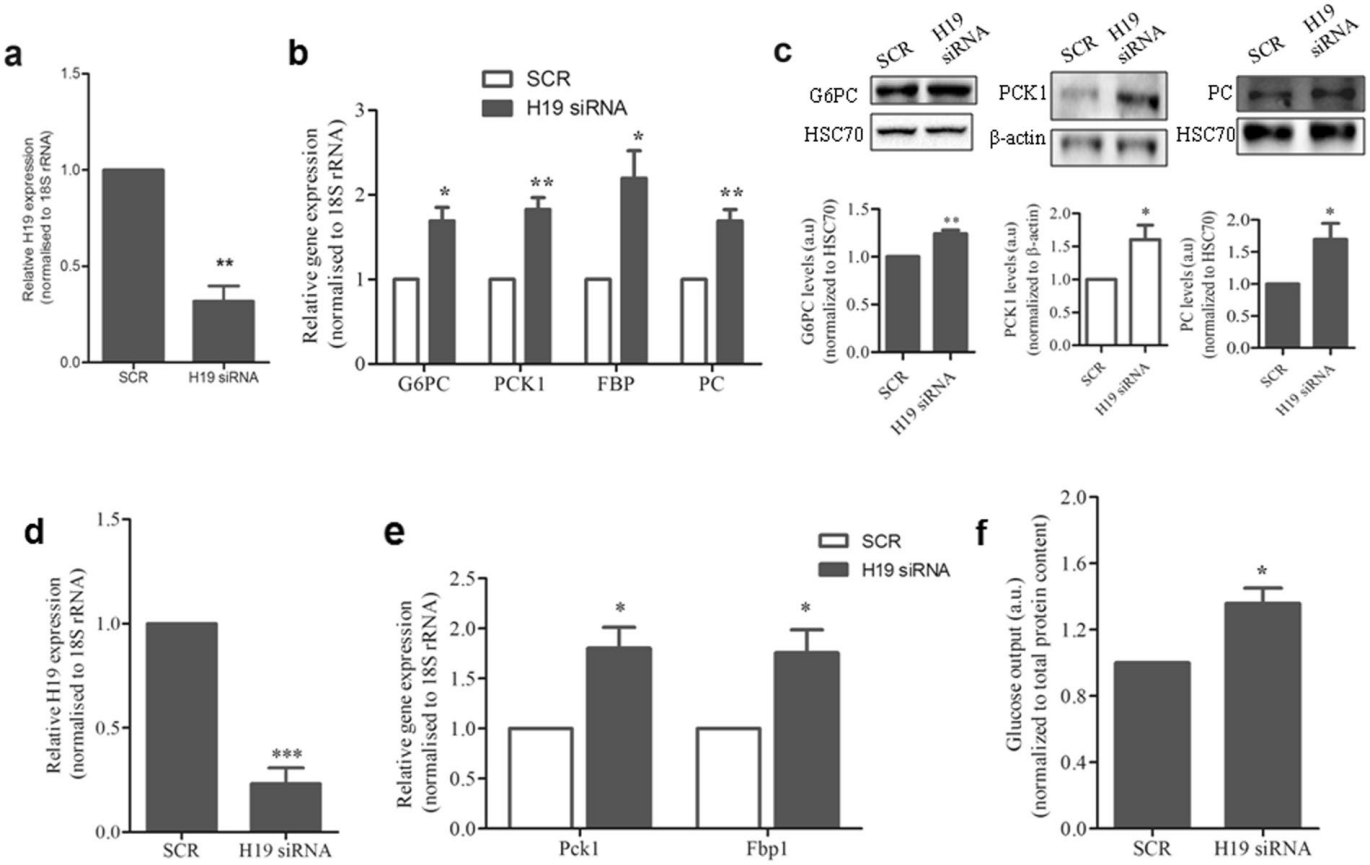
H19 inhibition elevates gluconeogenic gene expression. (**a**) HepG2 cells were reverse transfected with H19 siRNA (5 nM) and control cells were transfected with the scramble (SCR). After 48 h of incubation, RNA was isolated and assessed for the transcript level of H19 by qRT-PCR using specific primers. (**b**) RNA from HepG2 cells transfected with either H19 siRNA (5 nM) or the scramble was assessed for the expression of gluconeogenic genes (G6PC, PCK1, PC and FBP) by qRT-PCR using specific primers listed in Supplementary Table S3. (**c**) HepG2 cells were reverse transfected as in (**a**) and lysed after 48 h. 30 µg protein was probed for the detection of G6PC, PC and PCK1 by western blot analysis. HSC70 and β-actin were used as loading controls. Full length blots are presented in Supplementary Information (Supplementary Fig. S2). (**d**) Primary mouse hepatocytes were transfected with either the scramble or H19 siRNA (5 nM). After 48 h of incubation, RNA was isolated and H19 transcript levels were assessed by qRT-PCR using specific primers. (**e**) RNA from mouse primary hepatocytes which were transfected with either the scramble or H19 siRNA (5 nM, 48 h) was assessed for the expression of gluconeogenic genes (Pck1, Fbp1) by qRT-PCR using specific primers as listed in Supplementary Table S3. 18S rRNA was used as a normalization control for all the genes. (**f**) HepG2 cells were reverse transfected as in (**a**) and on termination of incubation, glucose output in the media was measured as described in the “Methods” section. Total protein content from whole cell lysates was used for normalization. Each experiment was done at least thrice and data is presented as mean ± s.e.m. *P < 0.05, **P < 0.01, ***P < 0.001 as compared to scramble transfected cells (SCR).

Liver plays a central role in regulating blood glucose homeostasis by maintaining a balance between uptake, storage and release of glucose. Hepatic gluconeogenesis majorly governs and maintains blood glucose levels by producing glucose in the liver and its uncontrolled activation leads to hyperglycemia. We further assessed the effect of H19 downregulation on the expression of key gluconeogenic genes. H19 levels were inhibited in HepG2 cells using specific siRNA (1–50 nM), and an optimum decrease of H19 levels was observed at 5 nM (Supplementary Fig. S1, Fig. 5a). Transient downregulation of the lncRNA H19 (5 nM, 48 h) resulted in a significant increase of almost 1.5 to 2 fold in the transcript levels of key gluconeogenic genes, G6PC (Glucose-6-Phosphatase), PCK1 (Phosphoenolpyruvate Carboxykinase 1) and PC (Pyruvate Carboxylase) and more than two fold in the levels of FBP (Fructose-1,6-Bisphosphatase 1) (Fig. 5b). As in the transcript levels, protein levels of G6PC, PCK1 and PC also showed a significant increase on inhibiting H19 levels in HepG2 cells as compared to the scramble (Fig. 5c). This was further validated in primary mouse hepatocytes where H19 siRNA (5 nM, 48 h) significantly inhibited cellular H19 levels (Fig. 5d). There was a significant increase of  ~1.8 fold in the transcript levels of Pck1 and Fbp1 genes (Fig. 5e). Pcx (pyruvate carboxylase) also showed a modest but non-significant increase in the expression (data not shown).

Such increase in levels of gluconeogenic genes were also observed in the db/db mice liver (Fig. 4b) and have been reported previously^13, 33^. This suggests that decreased hepatic H19 levels also possibly regulate gluconeogenic gene expression in *vivo*. Since increased hepatic gluconeogenesis is reflected by an increase in hepatic glucose output, we evaluated the effect of H19 downregulation on hepatic glucose output. There was a significant increase in the hepatic glucose output (Fig. 5f) during H19 inhibition suggesting that by altering gluconeogenesis, H19 also modulates glucose output from the liver.

### LncRNA H19 inhibition promotes nuclear localization of FoxO1

**Figure 6:**
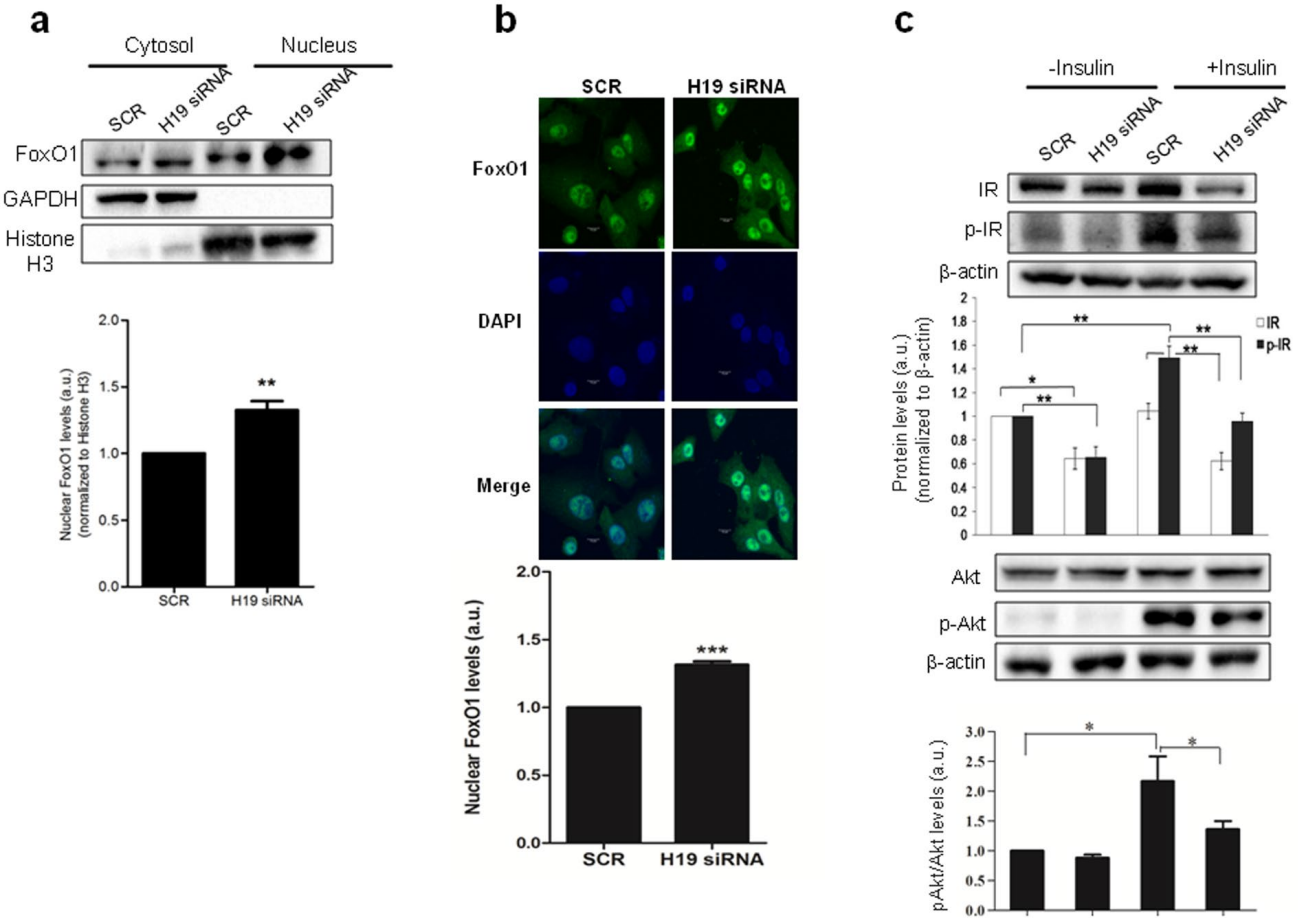
H19 inhibition increases nuclear localization of FoxO1 and impairs insulin signalling. (**a**) HepG2 cells were reverse transfected either with H19 siRNA (5 nM) or with the scramble (SCR), and after 48 h of incubation nuclear and cytoplasmic protein fractions were isolated as described in the “Methods” section. 30 µg protein from cytoplasmic and nuclear fraction was subjected to Western blot analysis using anti-FoxO1 antibody. GAPDH and Histone H3 were used as normalization controls for cytoplasmic and nuclear fractions, respectively. A representative blot is shown. Densitometric analysis of FoxO1 levels in the nuclear fractions of scramble and H19 siRNA transfected cells is also shown. Full length blots are presented in Supplementary Information (Supplementary Fig. S3). (**b**) HepG2 cells were plated on cover slips and reverse transfected either with H19 siRNA (5 nM) or the scramble (SCR) as described in “a”. After 48 h, cells were fixed and stained as described in the methods section with anti-FoxO1 antibody followed by Alexa Fluor-488 conjugated goat anti-rabbit IgG. Nuclei were counterstained with DAPI. Cells were visualized and levels of FoxO1 were quantified. Scale bar 10 µm. A representative figure is shown on the top and given below is the quantification of the nuclear levels of FoxO1. (**c**) HepG2 cells were transfected as in “a” and then incubated in the absence or presence of insulin (100 nM, 20 min). Cells were lysed, and the levels of total IR, pIR, total Akt and pAkt were estimated by Western blot analyses. β-Actin was taken as the normalization control. Full length blots are presented in Supplementary Information (Supplementary Fig. S4). Each value is the mean of three such experiments. Values presented are mean ± SEM. *P < 0.05, **P < 0.01, ***P < 0.001. a.u., arbitrary units.

Gluconeogenesis is a tightly regulated process controlled by a diverse array of transcription factors and regulators. Fork-head box protein O1 (FoxO1) is a key transcription factor regulating hepatic gluconeogenic genes^34^. The transcriptional activity of FoxO1 is dependent on its subcellular localization, as it shuttles between the cytoplasm and the nucleus due to precisely orchestrated post translational modifications^35^. Transcriptional activation of gluconeogenic genes due to increased nuclear presence of FoxO1 is frequently associated with increased gluconeogenesis^36^. We, therefore studied the effect of H19 inhibition on FoxO1 subcellular localization. There was a general increase in FoxO1 protein levels in the nuclear extract as compared to cytoplasmic extract which suggested increased nuclear retention of FoxO1 in HepG2 cells even under basal conditions (Fig. 6a). Interestingly, nuclear levels of FoxO1 significantly increased in H19 siRNA transfected HepG2 cells as compared to scramble siRNA transfected cells (Fig. 6a), suggesting that H19 inhibition promotes nuclear retention of FoxO1, which might be responsible for increasing gluconeogenic genes, transcription. We further validated the nuclear presence of FoxO1 by immunofluoresence and as in the Western Blot, there was increased presence of FoxO1 in the nucleus of H19 siRNA transfected HepG2 cells (Fig. 6b). Collectively, these results indicate towards a major role of H19 downregulation on increasing gluconeogenic genes, expression and glucose output by facilitating nuclear retention of FoxO1.

### H19 depletion regulates insulin signaling

Since we observed an increased presence of FoxO1 in the nucleus in the absence of H19, we now sought to assess the reasons for such an observation. Insulin signaling has been widely reported to exert effects on the nuclear and cytosolic presence of FoxO1^35^. We, therefore, evaluated the effects of H19 inhibition on the basal and insulin stimulated levels of insulin signaling intermediates. Inhibition of H19 levels using H19 siRNA significantly inhibited the levels of the insulin receptor (IR) and this was evident both, in the basal and insulin stimulated states (Fig. 6c). Such an inhibition in IR levels by H19 down-regulation was previously observed in C3H myotubes^19^. The basal levels of phosphorylated IR (p-IR) also significantly decreased in the presence of H19 siRNA. However, such H19 mediated decrease in p-IR levels was more pronounced in HepG2 cells stimulated with insulin (Fig. 6c). Further downstream, the levels of phosphorylation of Akt (p-AKt) also significantly decreased in the presence of H19 siRNA (Fig. 6c).

These results suggest that H19 inhibition attenuates insulin signaling, both at basal and insulin stimulated conditions. This, possibly is one of the reasons for the increased nuclear presence of FoxO1, although there might be other contributing events that are worthy of investigations.

## Discussion

Long noncoding RNAs are emerging as important regulatory factors of cellular processes within the body. Specifically, studies have demonstrated the roles of lncRNAs in regulating metabolic processes within the pancreas, liver, skeletal muscle and adipose tissue, deregulation of which lead to insulin resistance and diabetes^6, 19–23^. A recent study demonstrated the effect of lncRNA TUG1 on insulin secretion and apoptosis in mouse pancreatic β-cells. *In vivo* knockdown of TUG1 induced pancreatic β-cell apoptosis and decreased serum insulin and increased fasting blood glucose levels^37^. Decreased levels of a recently identified liver enriched lncRNA, lncLSTR has been associated with enhanced triglycerides (TG) clearance and decreased blood glucose in mice but further studies are required to explore how it regulates glucose metabolism^20^.

In the present study, using RNA sequencing we identify differentially expressed genes in the livers of db/db mice. Amongst the 218 altered genes in the db/db mice liver, there were three lncRNAs that were downregulated and of these, H19 was the most highly differentially expressed lncRNA. H19 is a long noncoding RNA, strongly expressed during mouse embryogenesis in the embryonic mesoderm and endoderm derived tissues and gets majorly repressed after birth^28^. It is located within a highly conserved imprinted H19-Igf2 locus where these two genes are reciprocally imprinted. Maternal diabetes alters methylation of the H19-Igf2 imprinting control region which affects fetal development, increases fetal mortality and decreases birth weight by altering the expression of H19-Igf2 genes^38^. Intrauterine hyperglycemia also decreases the expression of H19 and Igf2 in mouse pancreatic islets of F2 generation offsprings of gestational diabetes mellitus (GDM) mice. This is primarily due to abnormal methylation of the H19-Igf2 locus, which further induces transgenerational glucose intolerance and abnormal insulin levels^39^. Interestingly, decreased H19 levels have also been reported in the skeletal muscle of type 2 diabetes patients and insulin resistant rodents where H19 depletion results in impaired insulin signaling and decreased glucose uptake^19^. However, using Illumina’s HumanHT-122 Expression BeadChip Nilsson *et al*., found increased hepatic H19 levels in diabetic subjects^40^. This discrepancy between this study and our data may be due to the fact that the diabetic subjects taken for the study by Nilsson *et al*., were on oral antidiabetic medication, insulin treatment or both. The effect of these agents on the expression of H19 and other genes could not be ruled out.

Our data suggest that H19 possibly regulates hepatic glucose metabolism by acting as a novel upstream regulator of gluconeogenic genes. In the db/db mouse liver, H19 and gluconeogenic genes demonstrate inverse patterns of expression, and using HepG2 cells and primary mouse hepatocytes, we demonstrate that H19 inhibition promotes an increase in the expression of gluconeogenic genes and this was associated with increased hepatic glucose output. Increased gluconeogenesis is a major hallmark during fasting and postprandial hyperglycemia as seen in livers of type 2 diabetic subjects^14^. Such increase in hepatic gluconeogenesis is almost always due to increased expression of gluconeogenic genes that is mediated by a complex interaction among several regulators and transcription factors^41^.

In addition, in the recent past, several studies demonstrate the role of microRNAs (miRNAs) in regulating gluconeogenesis and glucose output during diabetes. While miR-214 suppresses gluconeogenesis by targeting ATF4^42^, miR-33b regulates gluconeogenesis by targeting PCK1 and G6PC^43^. Increased hepatic miR-22-3p hepatic levels promote hepatic gluconeogenesis and glucose output and *in vivo* inhibition of this miRNA improves hyperglycemia, glucose and pyruvate tolerance^33^. Insulin plays a major role in the regulation of hepatic gluconeogenesis and glucose production and in case of hepatic insulin resistance, it is precisely the impairment of this regulated pathway that majorly contributes to increased glucose output.

FoxO1 is a master regulator of gluconeogenesis^34^ which undergoes Akt-mediated phosphorylation that determines its nucleo-cytoplasmic presence^35^. During decreased insulin signaling or insulin resistance, the decreased FoxO1 phosphorylation by Akt increases its nuclear presence that elevates gluconeogenesis and increases glucose release^44^. We present data to show that H19 inhibition in HepG2 cells increases nuclear localization of FoxO1 by inhibiting insulin signaling, which further increases the transcription of gluconeogenic genes and promotes glucose output. H19 inhibition has been shown to impair insulin signaling in muscle cells and consequently decrease insulin stimulated glucose uptake^19^. Our results suggest that decreased hepatic H19 levels during diabetes promote FoxO1 nuclear localization and increase gluconeogenesis. FoxO1 is also reported to undergo certain post translational modifications and protein-protein interactions which also modifies/alters the proteins’ activity^35^. Long noncoding RNAs can directly interact with proteins and regulate gene expression transcriptionally or post-transcriptionally^45, 46^. Therefore, another possibility might be that H19 regulates FoxO1 by interacting with it or its post translational modifiers. These possibilities need to be explored for other possible mechanisms that facilitate nuclear translocation of FoxO1 during H19 inhibition.

To conclude, our results identify H19 as a critical regulator of hepatic gluconeogenesis during diabetes and provide new insights into the roles of lncRNAs in metabolism.

## Methods

### Animal Experiments

10–12 week old male normal (C57BLKs-db/ + ) and diabetic (C57BLKs-db/db) mice were obtained from CSIR-Central Drug Research Institute, Lucknow, India. Normal (weighing 22.05 ± 1.59 g with blood glucose levels: 151 ± 27 mg/dl) and diabetic animals (weighing 46.3 ± 8 g with blood glucose levels: 463 ± 98 mg/dl) were maintained at a 12:12 h light-dark cycle at the CSIR-Institute of Genomics and Integrative Biology, New Delhi (India) and were given ad libitum access to food and water. Mice were euthanized and livers from both db/+ and db/db animals were rapidly isolated and stored in RNA later (Ambion, Life Technologies, CA, USA) at −80 °C until further use. All experiments and procedures were approved by the Institutional Animal Ethics Committee (IAEC) of CSIR-Institute of Genomics and Integrative Biology, New Delhi, India and were according to the guidelines of the Committee for the Purpose of Control and Supervision of Experiments on Animals (CPCSEA), New Delhi, India.

### RNA Isolation and RNA Sequence Library Preparation and Data Generation

Total RNA from liver tissues of normal and diabetic mice was isolated using the Ambion mirVana isolation kit (Ambion®, Austin, TX, USA) and quantified using Infinite 200 PRO plate reader (Tecan, MANNEDORF, Switzerland). The 260/280 ratio were between 1.9–2.0. 1 µg of total RNA was used for RNA sample preparation using Truseq stranded RNA sample preparation kit along with Ribo-Zero Gold rRNA removal module according to manufacturer’s instructions (Illumina Inc., CA, USA). Briefly, lock nucleic acid based rRNA probes were used to remove ribosomal RNA and residual RNA was randomly fragmented at elevated temperature in the presence of divalent cations. Fragmented RNA was used to synthesize first strand cDNA using random hexamers and Superscript Reverse Transcriptase followed by second strand synthesis in the presence of actinomycin D and dUTP to enhance RNA dependent synthesis and strand specificity. Barcoded sequencing adapters were ligated to both the ends of the double stranded cDNA to facilitate multiplexed sequencing by synthesis using standard v.3 101 bp paired end sequencing on Hiseq. 2500 platform (Illumina Inc., CA, USA).

### RNA Sequencing Data Analysis

The sequencing reads obtained from normal (C57BLKs-db/+) and diabetic (C57BLKs-db/db) mice (n = 2) were adapter-trimmed along with a quality cut-off of Q20 and a minimum length threshold of 35 bases using Trimmomatic^47^. The reads were mapped to the reference genome GRCm38/mm10 using Tophat2^48^ that resulted in an average alignment rate close to 66%. We then used Cuffdiff^49^ to determine the differential expression of all known mouse genes between normal and diabetic mice using the transcript model from GENCODE (M3 version). Raw data of the sequenced reads from normal and diabetic mice livers have been deposited in the SRA with a series number SRP073480. R was subsequently used to generate a heatmap to represent the expression pattern of genes in the normal and diabetic mice liver. Pathway enrichment analysis for differentially expressed genes was performed with WikiPathways ontologies using EnrichR.

### Correlation Analyses and Pathway Annotation Network Analysis

The Pearson correlation of the expression profiles of H19 with all genes was performed using the R package, psych^50^. The pathway annotation network analysis of the correlated genes was conducted and visualized using the CytoScape plugin, ClueGO^51^.

### Cell Culture and Transfections

HepG2 cells were maintained in DMEM (Sigma, St. Louis, USA), supplemented with 10% Fetal Bovine Serum (Gibco, USA) and Antibiotic/Antimycotic (Gibco, USA). Endogenous levels of the lncRNA, H19 were knocked down by reverse transfection with the H19 siRNA (1–50 nM, GE Dharmacon, USA) and control cells were transfected with scramble (SCR) using Lipofectamine RNAiMax (Invitrogen, CA, USA). After an overnight incubation, the culture medium was replaced with fresh DMEM and 48 h later, RNA was isolated for further experiments.

### Primary mouse hepatocyte culture

Primary mouse hepatocytes were purchased from Cell Biologics, Inc. (Chicago, USA) and were grown on six-well plates (Corning CellBIND, NY, USA) in mouse hepatocyte medium (Cell Biologics, Inc., Chicago, USA) containing insulin, hydrocortisone, glutamine and epidermal growth factor as described in the manufacturer’s instructions. On attaining confluencency cells were transfected with either the scramble or the H19 siRNA (5 nM, 48 h, GE Dharmacon, USA) as described above. After 48 h, RNA was isolated from the cells using Trizol as described below and cDNA was synthesized by reverse transcribing 500 ng RNA (cells or tissues) using random hexamers and transcript levels of H19, Pck1, Pcx and Fbp1 were quantified by qRT-PCR using specific primers (Supplementary Table S3). 18SrRNA was used as a normalization control.

### RNA Isolation and qRT-PCR

Total RNA was isolated from cells transfected with either the scramble or H19 siRNA using TriZol (Invitrogen, CA, USA) according to manufacturer’s instructions. cDNA was synthesized by reverse transcribing 1 µg RNA (cells or tissues) using random hexamers and transcript levels of genes namely H19, Igf2 (Insulin-like growth factor 2), PCK1, G6PC, FBP and PC were quantified by qRT-PCR using specific primers (Supplementary Table S3) and Applied Biosystems SYBR Green Master mix (Life Technologies) in an Applied Biosystems Step One Plus Real Time PCR (Life Technologies). β-actin and 18S rRNA was used as a normalization control in tissues and cells, respectively. cDNA for miR-675 was prepared using specific stem-loop RT primers and U6 was used as a normalization control. Specificity of the reaction was verified by melt curve analysis and data was analyzed with threshold cycle (Ct) values using 2^−∆∆Ct^ method.

### Glucose Production Assay

HepG2 cells were transfected with the scramble or with H19 siRNA (5 nM, 48 h). Prior to completion of incubation, cells were serum starved for 2 h and then incubated in glucose production media (DMEM-glucose free, 20 mM sodium lactate, 2 mM pyruvate, and 0.5% BSA) for 4 h. Glucose concentrations in the media were measured using an Amplex Red Glucose/Glucose Oxidase Assay Kit (Life Technologies, CA, USA) and normalized to the total protein content of cells.

### Western Blotting

HepG2 cells were transfected with H19 siRNA (5 nM, 48 h) or with the scramble (control). Cells were lysed in RIPA Buffer (Sigma, St. Louis, USA) containing protease inhibitor (Calbiochem, Darmstadt, Germany). Protein samples (30 µg) were analyzed by Western Blotting for G6PC (Abcam, Cambridge, U.K.), PC (Sigma-Aldrich, St. Louis, MO) and PCK1 (Abcam, Cambridge, U.K.). HSC70 and β-actin were used as loading controls. For studying the nuclear and cytoplasmic localization, scramble or H19 siRNA transfected HepG2 cells were pelleted, washed with phosphate buffer saline (PBS) and lysed in cell lysis buffer (10 mM Hepes pH 7.9, 9 mM KCl, 1.5 mM MgCl_2_, NP-40, 0.1 mM EGTA, 0.1 mM DTT, 0.1 µM EDTA). Samples were then subjected to centrifugation at 7500 rpm for 15 min at 4 °C to collect the cytoplasmic fraction in the supernatant while the pellet was again washed with PBS and centrifuged at 7500 rpm for 5 min at 4 °C. The pellet obtained was re-suspended in nuclei lysis buffer (20 mM Hepes, 5 mM NaCl, 1.5 mM MgCl_2_, 0.1 mM EGTA, 0.1 mM EDTA, 1 mM DTT, 25% glycerol), centrifuged at 16000 rpm for 45 min at 4 °C and the supernatant was used as the nuclear fraction. Both cytoplasmic and nuclear fractions (30 µg each) were analyzed by western blotting for FoxO1 (Abcam, Cambridge, U.K.) by detecting with the ECL western blotting kit (Pierce, Thermo Scientific, Rockford, IL, USA). GAPDH (Santa Cruz Biotechnology, Dallas, TX) and Histone H3 (Cell Signaling Technology, Danvers, MA) was used as loading control for cytoplasmic and nuclear fractions, respectively. For evaluating the effects of H19 on insulin signaling, HepG2 cells transfected with either the scramble or H19siRNA (5 nM) were serum starved for 12 h and incubated in the absence or presence of insulin (100 nM, 20 min). Cells were lysed in RIPA Buffer containing protease and phosphatase inhibitors (Calbiochem, Darmstadt, Germany) and 30 µg protein was subjected to Western Blot analyses to evaluate the levels of IR, p-IR, Akt and p-Akt (Cell Signaling Technology, Danvers, MA) and β-actin was used as the loading control.

### Immunofluorescence

HepG2 cells were plated onto sterilized cover-slips placed on six-well plates, and transfected with H19 siRNA (5 nM) or with the scramble (control). After 48 h incubation, cells were washed with PBS and fixed with paraformaldehyde (4%) for 20 min. Cells were then washed with PBS and permeabilized with 0.2% TritonX 100 for 15 min. Permeabilized cells were washed with PBS and blocked with 1% BSA followed by incubation with rabbit anti-FoxO1 antibody (1:100, Abcam, Cambridge, U.K.) overnight at 4 °C. After washing, cells were incubated with Alexa Fluor-488 conjugated goat anti-rabbit IgG (1:1000, Invitrogen, CA, USA) for 2 h, counterstained and mounted with DAPI (Invitrogen, CA, USA). Images were acquired using Leica TCS SP8 confocal microscope (Germany) and were analysed with the Leica Application suite X software (LAS X).

### Densitometric Analysis

Densitometric analysis was done using Alpha DigiDoc 1201 software (Alpha Innotech Corporation, CA, USA) where the intensity of each band was normalized to background.

### Statistical Analysis

All data were analyzed using the two-tailed Student’s t-test and values presented as mean ± s.e.m., *P* < 0.05 were considered statistically significant.

## Electronic supplementary material

Supplementary information
