# Supplementary material for: RNA sequencing of db/db mice liver identifies lncRNA H19 as a key regulator of gluconeogenesis and hepatic glucose output
Source: Sci Rep. 2017 Aug 16;7:8312. doi: 10.1038/s41598-017-08281-7 (PMC5559625; doi:10.1038/s41598-017-08281-7)

Supplementary Data

RNA sequencing of db/db mice liver identifies lncRNA H19 as a key regulator of gluconeogenesis and hepatic glucose output

Neha Goyal[1,4], Ambily Sivadas [1,3,4], Shamsudheen KV [1,2,4], Rijith Jayarajan [1,2], Ankit Verma [1,2], Sridhar Sivasubbu[1,2,4], Vinod Scaria [1,3,4] & Malabika Datta [1,4]

[1]CSIR-Institute of Genomics and Integrative Biology, Mall Road, Delhi 110007, India;
[2]Genomics and Molecular Medicine, CSIR-Institute of Genomics and Integrative Biology, Mathura Road, Delhi 110025, India;
[3]GN Ramachandran Knowledge Centre for Genome Informatics, CSIR-Institute of Genomics and Integrative Biology, Mathura Road, Delhi 110025, India;
[4]Academy of Scientific and Innovative Research, Training and Development Complex, CSIR Campus, CSIR Road, Taramani, Chennai, India.

Address Correspondence:,

Dr. Malabika Datta
CSIR-Institute of Genomics and Integrative Biology
Mall Road, Delhi-110 007, India
Ph.: +91 11 27667439, 27667602 Ext 135
Fax: +91 11 27667471
e-mail: mdatta@igib.res.in

Supplementary Table S1 - RNA-Seq data summary and reference mapping.

	db/+		db/db	
	**C1**	**C2**	**D1**	**D2**
**Raw reads (x2)**	35.5M	34M	31.4M	42.8M
**Post-QC (x2)**	28.9M	26.8M	24.6M	33.8M
**Aligned reads (x2)**	19.8M (65.5%)	17.4M (62%)	17.3M (68%)	24M (69%)
**Expressed Genes** [GENCODE (M3) Catalog - FPKM ≥ 1]	10357	10443	10215	10292

Supplementary Table S2 - FPKM values of 218 differentially expressed genes

gene_id	gene	locus	value_1	value_2	log2(fold_change)	p_value	q_value	significant
ENSMUSG00000007122.6	Casq1	chr1:172209893-172219868	3.26148	0	-100	5.00E-05	0.0047	yes
ENSMUSG00000027022.8	Xirp2	chr2:67446001-67526614	1.30011	0	-100	5.00E-05	0.0047	yes
ENSMUSG00000033044.7	Dhrs7c	chr11:67798186-67816005	1.52324	0	-100	5.00E-05	0.0047	yes
ENSMUSG00000053093.10	Myh7	chr14:54968677-54994634	5.65554	0	-100	5.00E-05	0.0047	yes
ENSMUSG00000093861.1	Igkv1-110	chr6:68270526-68271267	1.72126	0	-100	0.00015	0.01175	yes
ENSMUSG00000094164.1	Ighv2-3	chr12:113611183-113611617	1.06254	0	-100	0.0007	0.0406	yes
ENSMUSG00000095335.1	Igkv3-5	chr6:70663297-70663895	1.26858	0	-100	0.0002	0.01507	yes
ENSMUSG00000095351.1	Igkv3-2	chr6:70698467-70699067	1.56291	0	-100	5.00E-05	0.0047	yes
ENSMUSG00000096422.1	Igkv12-44	chr6:69814630-69815100	1.31846	0	-100	0.00025	0.01837	yes
ENSMUSG00000051747.9	Ttn	chr2:76703979-76982547	13.2952	0.02988	-8.7973	5.00E-05	0.0047	yes
ENSMUSG00000021798.8	Ldb3	chr14:34526698-34588681	3.96291	0.01906	-7.6999	0.00045	0.02942	yes
ENSMUS	Mup7	chr4:600	24.0521	0.63716	-5.2384	0.0001	0.00851	yes

G00000073842.5		66468-60070475						
ENSMUSG00000020475.3	Pgam2	chr11:5801639-5803733	10.0409	0.38471	-4.706	5.00E-05	0.0047	yes
ENSMUSG00000032648.9	Pygm	chr19:6384398-6398459	9.624	0.42103	-4.5147	5.00E-05	0.0047	yes
ENSMUSG00000060600.10	Eno3	chr11:70657201-70662513	11.7899	0.51858	-4.5069	5.00E-05	0.0047	yes
ENSMUSG00000030246.6	Ldhb	chr6:142490248-142507957	5.8243	0.28253	-4.3656	0.0006	0.03678	yes
ENSMUSG00000068699.7	Flnc	chr6:29433152-29461888	2.21244	0.11179	-4.3068	5.00E-05	0.0047	yes
ENSMUSG00000028464.11	Tpm2	chr4:43514710-43523765	31.7284	1.68771	-4.2326	5.00E-05	0.0047	yes
ENSMUSG00000000031.10	H19	chr7:142575528-142578143	7.56898	0.41502	-4.1889	5.00E-05	0.0047	yes
ENSMUSG00000076612.2	Ighg2c	chr12:113285324-113288930	10.2257	0.62101	-4.0414	5.00E-05	0.0047	yes
ENSMUSG00000026208.9	Des	chr1:75360328-75375012	13.1205	0.95265	-3.7837	5.00E-05	0.0047	yes
ENSMUSG00000057722.12	Lepr	chr4:101717403-101815352	6.39182	0.51362	-3.6375	5.00E-05	0.0047	yes
ENSMUSG00000064179.8	Tnnt1	chr7:4504569-4516382	7.42753	0.66772	-3.4756	5.00E-05	0.0047	yes
ENSMUSG00000006221.7	Hspb7	chr4:141420778-141425311	1.61609	0.14597	-3.4688	0.00045	0.02942	yes
ENSMUSG0000007	Hsp25-ps1	chr13:45078497-	8.58126	0.7788	-3.4619	0.00055	0.03425	yes

8915.1		4508122 2						
ENSMUS G0000003 1791.7	Tmem38 a	chr8:725 72102- 7258728 2	3.32143	0.30398	-3.4498	0.00015	0.01175	yes
ENSMUS G0000002 9752.7	Asns	chr6:767 5168- 7693254	4.1332	0.38163	-3.437	5.00E-05	0.0047	yes
ENSMUS G0000002 6950.11	Neb	chr2:521 36646- 5233879 8	13.3842	1.3092	-3.3538	5.00E-05	0.0047	yes
ENSMUS G0000003 1633.4	Slc25a4	chr8:462 06796- 4621128 4	19.4112	1.96401	-3.305	5.00E-05	0.0047	yes
ENSMUS G0000007 6614.2	Ighg1	chr12:11 3328975- 1133305 23	6.4415	0.66061	-3.2855	0.00015	0.01175	yes
ENSMUS G0000007 6613.2	Ighg2b	chr12:11 3306388- 1133079 33	12.2121	1.28796	-3.2452	5.00E-05	0.0047	yes
ENSMUS G0000005 3303.9	Slc22a26	chr19:77 81040- 7802667	28.2167	3.15445	-3.1611	5.00E-05	0.0047	yes
ENSMUS G0000000 4951.10	Hspb1	chr5:135 887918- 1358895 63	4.56751	0.54447	-3.0685	0.00035	0.02465	yes
ENSMUS G0000002 7796.2	Smad9	chr3:547 55581- 5480125 7	1.58131	0.18851	-3.0684	5.00E-05	0.0047	yes
ENSMUS G0000002 7499.7	Pkia	chr3:736 6603- 7445366	1.18254	0.15031	-2.9759	0.0006	0.03678	yes
ENSMUS G0000007 6609.1	Igkc	chr6:707 26434- 7072675 7	51.5235	6.90192	-2.9002	5.00E-05	0.0047	yes
ENSMUS G0000003 2060.9	Cryab	chr9:507 52757- 5075663 3	7.74312	1.0729	-2.8514	5.00E-05	0.0047	yes
ENSMUS G0000001 8566.9	Slc2a4	chr11:69 942538- 6994818	1.94366	0.27801	-2.8056	0.00065	0.03884	yes

		8						
ENSMUSG00000067149.4	Igj	chr5:88520511-88527891	3.49652	0.50702	-2.7858	0.0001	0.00851	yes
ENSMUSG00000027533.10	Fabp5	chr3:10012547-10016607	7.38389	1.07853	-2.7753	5.00E-05	0.0047	yes
ENSMUSG00000023092.11	Fhl1	chrX:56731786-56793346	9.2289	1.38352	-2.7378	5.00E-05	0.0047	yes
ENSMUSG00000095079.1	Igha	chr12:113258767-113260236	10.8879	1.66857	-2.706	5.00E-05	0.0047	yes
ENSMUSG00000074254.3	Cyp2a4	chr7:26307189-26315088	80.2433	12.555	-2.6761	5.00E-05	0.0047	yes
ENSMUSG00000074280.5	Gm6166	chr9:57483912-57484585	9.31749	1.48589	-2.6486	0.00015	0.01175	yes
ENSMUSG00000073834.5	Mup11	chr4:60658465-60662411	80.4653	13.0867	-2.6203	5.00E-05	0.0047	yes
ENSMUSG00000029417.8	Cxcl9	chr5:92321330-92328079	6.1194	1.07663	-2.5069	5.00E-05	0.0047	yes
ENSMUSG00000000628.5	Hk2	chr6:82725024-82774454	2.13531	0.45014	-2.246	5.00E-05	0.0047	yes
ENSMUSG00000032366.10	Tpm1	chr9:67022589-67049406	39.7432	9.0572	-2.1336	5.00E-05	0.0047	yes
ENSMUSG00000090877.3	Hspa1b	chr17:34956435-34959238	5.8126	1.3632	-2.0922	5.00E-05	0.0047	yes
ENSMUSG00000050440.7	Hamp	chr7:30942370-30944017	356.133	92.4839	-1.9451	5.00E-05	0.0047	yes
ENSMUS	Cyp2c40	chr19:39	27.3123	7.36805	-1.8902	0.00055	0.03425	yes

G00000025004.10		767070-39812814						
ENSMUSG00000024962.8	Vegfb	chr19:6982472-6987651	6.20312	1.7005	-1.867	0.0001	0.00851	yes
ENSMUSG00000040026.7	Saa3	chr7:46711997-46715676	21.0316	5.83095	-1.8508	5.00E-05	0.0047	yes
ENSMUSG00000015568.10	Lpl	chr8:68880490-68907448	9.96153	2.81926	-1.8211	5.00E-05	0.0047	yes
ENSMUSG00000072849.5	Serpina1e	chr12:103946930-103958975	463.248	133.432	-1.7957	0.00065	0.03884	yes
ENSMUSG00000036854.9	Hspb6	chr7:30539133-30559838	7.99937	2.37815	-1.7501	0.0009	0.04797	yes
ENSMUSG00000092274.2	Neat1	chr19:5824707-5845478	8.72721	2.7236	-1.68	5.00E-05	0.0047	yes
ENSMUSG00000042834.9	Nrep	chr18:33437018-33464029	4.85936	1.53699	-1.6607	0.0002	0.01507	yes
ENSMUSG00000039519.6	Cyp7b1	chr3:18071949-18243338	17.023	5.43518	-1.6471	0.0002	0.01507	yes
ENSMUSG00000092008.1	Cyp2c69	chr19:39842659-39886769	4.83307	1.54436	-1.6459	0.0003	0.02164	yes
ENSMUSG00000039323.13	Igfbp2	chr1:72824502-72852474	189.748	60.638	-1.6458	5.00E-05	0.0047	yes
ENSMUSG00000056978.6	Hamp2	chr7:30922371-30924181	79.7169	25.6126	-1.638	5.00E-05	0.0047	yes
ENSMUSG00000022218.10	Tgm1	chr14:55700008-55713492	5.63318	1.91252	-1.5585	5.00E-05	0.0047	yes
ENSMUS	Ar	chrX:981	1.45873	0.51708	-1.4962	0.0001	0.00851	yes

G00000046532.7		49720-98323215						
ENSMUSG00000036594.9	H2-Aa	chr17:34282743-34287827	12.0925	4.45341	-1.4411	0.0003	0.02164	yes
ENSMUSG00000032898.6	Fbxo21	chr5:117976769-118010191	15.1085	5.59704	-1.4326	5.00E-05	0.0047	yes
ENSMUSG00000022181.10	C6	chr15:4727174-4815456	20.4765	7.6384	-1.4226	0.00085	0.04571	yes
ENSMUSG00000025197.8	Cyp2c44	chr19:44005021-44029247	84.4544	31.7178	-1.4129	5.00E-05	0.0047	yes
ENSMUSG00000019838.10	Slc16a10	chr10:40033534-40142254	20.981	8.06433	-1.3795	5.00E-05	0.0047	yes
ENSMUSG00000037266.13	Rsrp1	chr4:134923591-134927671	24.5877	9.49961	-1.372	5.00E-05	0.0047	yes
ENSMUSG00000033065.8	Pfkm	chr15:98038743-98132447	10.5786	4.18554	-1.3377	0.00045	0.02942	yes
ENSMUSG00000060807.7	Serpina6	chr12:103646629-103657212	151.363	60.4092	-1.3252	5.00E-05	0.0047	yes
ENSMUSG00000024610.9	Cd74	chr18:60803847-60812646	59.5103	24.2748	-1.2937	5.00E-05	0.0047	yes
ENSMUSG00000100094.1	RP23-181K4.2	chr7:65803110-65806449	48.0523	20.1054	-1.257	0.0001	0.00851	yes
ENSMUSG00000008035.7	Mid1ip1	chrX:10707175-10719690	43.698	18.4455	-1.2443	0.0008	0.04444	yes
ENSMUSG00000073421.5	H2-Ab1	chr17:34263208-34269418	10.1955	4.31018	-1.2421	0.0007	0.0406	yes

ENSMUSG00000031441.10	Atp11a	chr8:12757013-13020905	7.27474	3.09555	-1.2327	0.00095	0.04952	yes
ENSMUSG00000039218.11	Srrm2	chr17:23790531-23829109	15.7919	6.85716	-1.2035	0.00085	0.04571	yes
ENSMUSG00000041698.6	Slco1a1	chr6:141907281-141946962	25.6429	11.4024	-1.1692	0.0002	0.01507	yes
ENSMUSG00000000303.7	Cdh1	chr8:106603350-106670246	4.81223	2.16504	-1.1523	0.00035	0.02465	yes
ENSMUSG00000038872.9	Zfhx3	chr8:108703099-108961630	1.65049	0.75542	-1.1275	0.00075	0.04266	yes
ENSMUSG00000011148.8	Adssl1	chr12:112620044-112641354	10.1818	4.70572	-1.1135	0.00065	0.03884	yes
ENSMUSG00000052837.5	Junb	chr8:84976908-84978748	10.9806	5.19914	-1.0786	0.00075	0.04266	yes
ENSMUSG00000025132.8	Arhgdia	chr11:120578103-120581624	21.5495	11.16	-0.9493	0.00085	0.04571	yes
ENSMUSG00000020307.9	Cdc34	chr10:79682194-79688394	18.6089	36.0997	0.95599	0.00085	0.04571	yes
ENSMUSG00000009646.8	Pla2g12b	chr10:59403659-59421976	16.5746	32.3678	0.96559	0.00095	0.04952	yes
ENSMUSG00000038150.7	Ormdl3	chr11:98581255-98587368	13.9366	27.6454	0.98816	0.0007	0.0406	yes
ENSMUSG00000038403.9	Hfe2	chr3:96525184-96529216	10.4075	20.7492	0.99543	0.00065	0.03884	yes
ENSMUSG0000002	Rtfdc1	chr2:172393793-	9.31587	19.0373	1.03107	0.00095	0.04952	yes

7502.6		1724699<br>08						
ENSMUS<br>G0000003<br>6752.4	Tubb4b	chr2:252<br>18744-<br>2522470<br>2	17.0668	35.1857	1.0438	0.00015	0.01175	yes
ENSMUS<br>G0000004<br>0562.7	Gstm2	chr3:107<br>981701-<br>1079864<br>53	10.7256	22.1266	1.04473	0.00055	0.03425	yes
ENSMUS<br>G0000005<br>0777.7	Tmem37	chr1:120<br>067376-<br>1200740<br>74	18.9585	40.5323	1.09623	0.00015	0.01175	yes
ENSMUS<br>G0000002<br>2742.5	Cpox	chr16:58<br>670207-<br>5868039<br>1	23.0178	49.4976	1.10461	0.0007	0.0406	yes
ENSMUS<br>G0000002<br>7222.9	Pex16	chr2:923<br>65045-<br>9238121<br>7	21.6163	46.8077	1.11462	0.00055	0.03425	yes
ENSMUS<br>G0000002<br>8383.12	Hsdl2	chr4:595<br>81562-<br>5961868<br>9	12.2322	26.4919	1.11487	0.00055	0.03425	yes
ENSMUS<br>G0000001<br>8585.9	Atox1	chr11:55<br>446640-<br>5546123<br>9	41.3268	89.5367	1.1154	0.00015	0.01175	yes
ENSMUS<br>G0000000<br>2289.10	Angptl4	chr17:33<br>773749-<br>3378157<br>5	24.9229	54.4419	1.12725	0.00045	0.02942	yes
ENSMUS<br>G0000003<br>2350.8	Gclc	chr9:777<br>54534-<br>7779448<br>5	24.483	55.1182	1.17075	0.0004	0.02704	yes
ENSMUS<br>G0000003<br>9886.3	Tmem12<br>0a	chr5:135<br>735484-<br>1357442<br>71	17.4149	39.5456	1.1832	0.0001	0.00851	yes
ENSMUS<br>G0000001<br>5337.5	Endog	chr2:301<br>71492-<br>3017845<br>9	15.5334	35.3518	1.18641	0.0009	0.04797	yes
ENSMUS<br>G0000002<br>0142.7	Slc1a4	chr11:20<br>302179-<br>2033271<br>3	1.16807	2.68144	1.19888	0.00095	0.04952	yes

ENSMUSG00000025260.5	Hsd17b10	chrX:152001844-152004442	57.7084	133.363	1.20851	0.0001	0.00851	yes
ENSMUSG00000024827.9	Gldc	chr19:30098448-30175418	23.1202	53.8621	1.22012	0.0004	0.02704	yes
ENSMUSG00000044469.7	Tnfaip8l1	chr17:56162476-56173955	2.49143	5.85408	1.23247	0.00015	0.01175	yes
ENSMUSG00000025059.11	Gyk	chrX:85701936-85776819	9.50386	22.3524	1.23384	0.0001	0.00851	yes
ENSMUSG00000047492.4	Inhbe	chr10:127349401-127351772	8.09466	19.0552	1.23514	5.00E-05	0.0047	yes
ENSMUSG00000024747.3	Aldh1a7	chr19:20692952-20727562	42.6169	100.641	1.23971	0.00015	0.01175	yes
ENSMUSG00000015357.5	Clpx	chr9:65294259-65330658	10.5995	25.0619	1.2415	0.0001	0.00851	yes
ENSMUSG00000038181.11	Chpf2	chr5:24586740-24602012	3.34433	7.91633	1.24311	0.00015	0.01175	yes
ENSMUSG00000022790.8	Igsf11	chr16:38901470-39027159	5.00998	11.8612	1.24338	0.00055	0.03425	yes
ENSMUSG00000036181.2	Hist1h1c	chr13:23738807-23740367	19.7177	46.6827	1.24339	5.00E-05	0.0047	yes
ENSMUSG00000039956.3	Mrap	chr16:90738323-90749785	12.4748	29.6024	1.2467	0.00015	0.01175	yes
ENSMUSG00000026614.6	Slc30a10	chr1:185447115-185468762	1.64241	3.90073	1.24793	5.00E-05	0.0047	yes
ENSMUSG0000000	Epha2	chr4:141301239-	1.94415	4.65885	1.26084	0.0005	0.03233	yes

6445.3		141329384						
ENSMUSG00000021336.11	Slc17a4	chr13:23897737-23915007	5.62269	13.4836	1.26188	5.00E-05	0.0047	yes
ENSMUSG00000029162.10	Khk	chr5:30921555-30931246	123.176	296.296	1.26632	0.0007	0.0406	yes
ENSMUSG00000032602.5	Slc25a20	chr9:108662097-108684641	28.7479	69.2966	1.26933	5.00E-05	0.0047	yes
ENSMUSG00000078650.2	G6pc	chr11:101367560-101377903	65.2522	157.482	1.27109	0.00095	0.04952	yes
ENSMUSG00000041845.5	Rhod	chr19:4425458-4477447	5.6321	13.7653	1.28929	0.00025	0.01837	yes
ENSMUSG00000038539.9	Atf5	chr7:44812256-44840809	32.7127	80.1186	1.29229	0.0002	0.01507	yes
ENSMUSG00000042632.11	Pla2g6	chr15:79286227-79328390	13.6229	33.5476	1.30017	0.00025	0.01837	yes
ENSMUSG00000042476.8	Abcb4	chr5:8893720-8959225	28.3657	70.5111	1.3137	5.00E-05	0.0047	yes
ENSMUSG00000036781.8	Rps27l	chr9:66946085-66949516	62.4468	155.978	1.32064	5.00E-05	0.0047	yes
ENSMUSG00000021751.8	Acox2	chr14:8225510-8259353	50.6442	127.465	1.33163	0.0004	0.02704	yes
ENSMUSG00000031762.6	Mt2	chr8:94172617-94173567	127.023	322.023	1.34207	0.0007	0.0406	yes
ENSMUSG00000047446.13	Arl4a	chr12:40005446-40038025	3.70304	9.51991	1.36224	0.0005	0.03233	yes
ENSMUSG00000037583.2	Nr0b2	chr4:133553375-1335565	6.94196	17.9273	1.36874	5.00E-05	0.0047	yes

		36						
ENSMUS G00000009633.2	G0s2	chr1:193272160-193273188	43.4076	112.309	1.37146	5.00E-05	0.0047	yes
ENSMUS G00000067279.2	Ppp1r3c	chr19:36731736-36736653	6.34858	16.4941	1.37744	5.00E-05	0.0047	yes
ENSMUS G00000063558.4	Aox1	chr1:58029930-58106413	7.2043	18.8961	1.39116	5.00E-05	0.0047	yes
ENSMUS G00000029630.10	Cyp3a25	chr5:145977193-146009618	84.2196	221.629	1.39592	0.0001	0.00851	yes
ENSMUS G00000056313.5	1810011 O10Rik	chr8:24422403-24440008	5.49141	14.4559	1.39641	5.00E-05	0.0047	yes
ENSMUS G00000060002.9	Chpt1	chr10:88452744-88504073	25.9204	68.2863	1.39751	5.00E-05	0.0047	yes
ENSMUS G00000023019.7	Gpd1	chr15:99715542-99725005	44.5475	118.698	1.41387	5.00E-05	0.0047	yes
ENSMUS G00000024778.7	Fas	chr19:34290658-34327770	3.94372	10.5494	1.41953	0.0008	0.04444	yes
ENSMUS G00000010755.11	Cars	chr7:143557229-143600090	5.02322	13.512	1.42755	5.00E-05	0.0047	yes
ENSMUS G00000034220.7	Gpc1	chr1:92820957-92860779	2.41414	6.58427	1.44751	5.00E-05	0.0047	yes
ENSMUS G00000067144.6	Slc22a7	chr17:46432184-46438477	8.22372	22.5326	1.45415	5.00E-05	0.0047	yes
ENSMUS G00000026890.14	Lhx6	chr2:36081952-36105408	0.61975	1.707	1.4617	0.00085	0.04571	yes
ENSMUS	Dmgdh	chr13:93	70.1197	194.31	1.47047	0.0003	0.02164	yes

G0000004 2102.6		674432-9375283 1						
ENSMUS G0000002 8766.5	Alpl	chr4:137 741732-1377963 84	6.31384	17.5613	1.47581	0.0002	0.01507	yes
ENSMUS G0000003 1775.4	Pllp	chr8:946 74894-9469624 2	1.87823	5.26222	1.4863	0.00025	0.01837	yes
ENSMUS G0000003 7440.7	Vnn1	chr10:23 894687-2390534 3	9.69456	27.2706	1.4921	5.00E-05	0.0047	yes
ENSMUS G0000004 1653.4	Pnpla3	chr15:84 167815-8418951 2	0.73123	2.06049	1.49458	0.00045	0.02942	yes
ENSMUS G0000007 2664.5	Ugt3a1	chr15:92 73318-9315032	11.7818	33.2059	1.49488	0.0008	0.04444	yes
ENSMUS G0000005 2565.6	Hist1h1d	chr13:23 555086-2355583 0	4.99362	14.1914	1.50685	0.0004	0.02704	yes
ENSMUS G0000003 0545.8	Pex11a	chr7:797 35956-7974313 1	8.64952	24.8737	1.52393	5.00E-05	0.0047	yes
ENSMUS G0000003 6957.7	Lrfn3	chr7:303 55513-3036277 2	0.8538	2.49728	1.54839	0.00035	0.02465	yes
ENSMUS G0000003 0762.6	Aqp8	chr7:123 462293-1234680 03	9.52634	27.9933	1.55509	5.00E-05	0.0047	yes
ENSMUS G0000002 0108.3	Ddit4	chr10:59 949674-5995177 0	3.19017	9.38872	1.5573	5.00E-05	0.0047	yes
ENSMUS G0000002 8655.6	Mfsd2a	chr4:122 946849-1229611 88	27.9293	82.5885	1.56416	5.00E-05	0.0047	yes
ENSMUS G0000001 6028.9	Celsr1	chr15:85 898757-8603377 7	0.66031	1.96241	1.5714	5.00E-05	0.0047	yes

ENSMUSG00000035372.1	1810055G02Rik	chr19:3708332-3717881	10.512	31.3618	1.57698	5.00E-05	0.0047	yes
ENSMUSG00000022010.14	Tsc22d1	chr14:76414960-76507765	8.89844	26.736	1.58716	5.00E-05	0.0047	yes
ENSMUSG00000011305.6	Plin5	chr17:56111600-56117596	8.89591	26.978	1.60057	5.00E-05	0.0047	yes
ENSMUSG00000060519.6	Tor3a	chr1:156653616-156674356	1.30593	4.06697	1.63887	0.00075	0.04266	yes
ENSMUSG00000036216.5	Leap2	chr11:53422180-53423170	53.0052	165.874	1.64589	5.00E-05	0.0047	yes
ENSMUSG00000048856.8	Slc25a47	chr12:108851128-108856814	139.028	435.486	1.64725	0.0001	0.00851	yes
ENSMUSG00000029269.6	Sult1b1	chr5:87513338-87538195	5.31234	16.735	1.65545	5.00E-05	0.0047	yes
ENSMUSG00000028715.2	Cyp4a14	chr4:115486199-115496142	120.639	380.482	1.65713	0.0004	0.02704	yes
ENSMUSG00000020326.7	Ccng1	chr11:40748551-40755311	5.52366	17.4392	1.65864	5.00E-05	0.0047	yes
ENSMUSG00000030609.13	Aen	chr7:78895853-78911209	2.17754	7.02138	1.68906	5.00E-05	0.0047	yes
ENSMUSG00000023057.5	Fabp2	chr3:122864736-122924116	8.60366	27.9748	1.7011	5.00E-05	0.0047	yes
ENSMUSG00000074264.7	Amy1	chr3:113555709-113630149	36.0277	118.48	1.71746	5.00E-05	0.0047	yes
ENSMUSG00000026853.10	Crat	chr2:30392253-3041581	8.69708	28.6125	1.71804	5.00E-05	0.0047	yes

		3						
ENSMUSG00000028494.7	Plin2	chr4:86648385-86670060	76.2897	252.323	1.72571	5.00E-05	0.0047	yes
ENSMUSG00000020423.6	Btg2	chr1:134075169-134079120	1.60436	5.44606	1.76322	5.00E-05	0.0047	yes
ENSMUSG00000042429.8	Adora1	chr1:134193447-134235431	1.57885	5.40014	1.77413	5.00E-05	0.0047	yes
ENSMUSG00000091867.1	Cyp2a22	chr7:26931630-26939386	7.87446	26.9767	1.77646	5.00E-05	0.0047	yes
ENSMUSG00000028893.8	Sesn2	chr4:132492031-132510501	1.06661	3.79133	1.82967	5.00E-05	0.0047	yes
ENSMUSG00000003555.7	Cyp17a1	chr19:46667164-46672974	25.5367	90.8675	1.83119	5.00E-05	0.0047	yes
ENSMUSG00000022883.10	Robo1	chr16:72663148-73046095	0.71879	2.64975	1.88222	5.00E-05	0.0047	yes
ENSMUSG00000029763.12	Exoc4	chr6:33249084-33973979	4.49249	17.1033	1.92869	5.00E-05	0.0047	yes
ENSMUSG00000032418.10	Me1	chr9:86581370-86701253	5.41139	20.7037	1.93582	5.00E-05	0.0047	yes
ENSMUSG00000041959.9	S100a10	chr3:93555079-93564643	27.1462	104.407	1.9434	5.00E-05	0.0047	yes
ENSMUSG00000023828.2	Slc22a3	chr17:12419971-12507704	0.59503	2.33171	1.97035	5.00E-05	0.0047	yes
ENSMUSG00000021226.7	Acot2	chr12:83987860-83993873	1.34747	5.286	1.97193	5.00E-05	0.0047	yes
ENSMUS	Hist1h4n	chr13:21	3.30177	13.6194	2.04435	0.00045	0.02942	yes

G0000006 9305.3		831766-2183219 6						
ENSMUS G0000006 8083.1	Cyp2d40	chr15:82 759834-8276418 3	27.3873	113.33	2.04895	5.00E-05	0.0047	yes
ENSMUS G0000005 1579.5	Tceal8	chrX:136 168983-1361723 42	4.74987	19.7275	2.05425	5.00E-05	0.0047	yes
ENSMUS G0000004 2041.6	2010003 K11Rik	chr19:44 96789-4498583	4.72342	19.6457	2.05631	5.00E-05	0.0047	yes
ENSMUS G0000002 0604.8	Arsg	chr11:10 9425945-1095733 30	1.87664	7.88634	2.0712	0.0004	0.02704	yes
ENSMUS G0000004 1567.3	Serpina1 2	chr12:10 4028768-1040444 43	0.29203	1.2467	2.09392	0.00075	0.04266	yes
ENSMUS G0000002 5003.6	Cyp2c39	chr19:39 510861-3956852 9	1.16725	5.03282	2.10825	5.00E-05	0.0047	yes
ENSMUS G0000009 6105.1	Bhmt-ps1	chr4:263 68983-2637020 7	5.1931	22.5453	2.11816	5.00E-05	0.0047	yes
ENSMUS G0000004 5776.2	Lrtm1	chr14:28 904942-2972186 4	0.70904	3.27268	2.20653	5.00E-05	0.0047	yes
ENSMUS G0000002 3073.1	Slc10a2	chr8:508 5622-5105232	0.98894	4.58736	2.21371	5.00E-05	0.0047	yes
ENSMUS G0000003 8754.4	Elovl3	chr19:46 131896-4613530 7	1.59146	7.74356	2.28265	5.00E-05	0.0047	yes
ENSMUS G0000000 2944.10	Cd36	chr5:177 81689-1788895 9	11.6802	57.3461	2.29563	5.00E-05	0.0047	yes
ENSMUS G0000000 3721.9	Insig2	chr1:121 304352-1213325 89	21.4696	110.427	2.36273	5.00E-05	0.0047	yes
ENSMUS	Tubb2a	chr13:34	4.16672	21.9011	2.39402	5.00E-05	0.0047	yes

G0000005 8672.6		074300-3407800 8						
ENSMUS G0000007 9494.1	Cml5	chr6:858 08023-8582097 2	1.81887	9.72126	2.4181	0.0001	0.00851	yes
ENSMUS G0000003 1886.8	Ces2e	chr8:104 926259-1049346 72	38.1577	204.236	2.42019	5.00E-05	0.0047	yes
ENSMUS G0000006 9324.5	Gm5096	chr18:87 756285-8775814 3	115.049	615.954	2.42058	5.00E-05	0.0047	yes
ENSMUS G0000000 6218.4	Fam131c	chr4:141 368219-1413841 75	0.87537	4.74553	2.4386	5.00E-05	0.0047	yes
ENSMUS G0000005 7933.5	Gsta2	chr9:783 31017-7835578 8	3.02156	16.5823	2.45628	5.00E-05	0.0047	yes
ENSMUS G0000006 1292.6	Cyp3a59	chr5:146 079257-1461132 85	7.07867	39.6305	2.48506	5.00E-05	0.0047	yes
ENSMUS G0000003 0643.8	Rab30	chr7:927 41602-9283711 7	1.10174	6.40984	2.5405	0.0004	0.02704	yes
ENSMUS G0000007 4768.5	Bhmt	chr13:93 616890-9363796 1	123.467	722.312	2.5485	5.00E-05	0.0047	yes
ENSMUS G0000004 9493.8	Pls1	chr9:957 52641-9584531 1	0.5191	3.14768	2.6002	0.0006	0.03678	yes
ENSMUS G0000006 6072.8	Cyp4a10	chr4:115 518263-1155336 49	25.9562	162.605	2.64722	5.00E-05	0.0047	yes
ENSMUS G0000003 8776.8	Ephx1	chr1:180 976154-1810209 04	61.0153	386.579	2.66352	5.00E-05	0.0047	yes
ENSMUS G0000002 1701.7	Plk2	chr13:11 0395043-1104008	1.38019	8.89865	2.68872	5.00E-05	0.0047	yes

		44						
ENSMUSG00000019935.11	Slc17a8	chr10:89574019-89621253	0.85826	5.93384	2.78948	5.00E-05	0.0047	yes
ENSMUSG00000072949.5	Acot1	chr12:84009501-84017670	3.11668	21.6708	2.79767	5.00E-05	0.0047	yes
ENSMUSG00000008153.6	Clstn3	chr6:124430758-124464794	0.40684	2.85848	2.81272	0.00015	0.01175	yes
ENSMUSG00000038768.8	9130409I23Rik	chr1:181051236-181060667	0.45883	4.21215	3.19854	5.00E-05	0.0047	yes
ENSMUSG00000030004.6	Nat8	chr6:85830387-85832082	0.65219	6.70661	3.36221	5.00E-05	0.0047	yes
ENSMUSG00000032080.6	Apoa4	chr9:46240695-46243529	37.9121	393.101	3.37417	5.00E-05	0.0047	yes
ENSMUSG00000024411.9	Aqp4	chr18:15389393-15403684	0.30136	3.30165	3.45363	5.00E-05	0.0047	yes
ENSMUSG00000029188.9	Slc34a2	chr5:53038081-53071664	0.12019	1.70108	3.82308	0.00085	0.04571	yes
ENSMUSG00000029272.7	Sult1e1	chr5:87575967-87591611	0.51403	7.89722	3.94141	0.00065	0.03884	yes
ENSMUSG00000038508.7	Gdf15	chr8:70629392-70632456	0.45748	7.28541	3.99322	5.00E-05	0.0047	yes
ENSMUSG00000041801.5	Phlda3	chr1:135766118-135769136	0.55524	9.89557	4.15559	5.00E-05	0.0047	yes
ENSMUSG00000075014.1	Gm10800	chr2:98666546-98667301	0.4364	35.7039	6.3543	0.00085	0.04571	yes
ENSMUS	Cdkn1a	chr17:29	0.29636	31.956	6.75261	0.0001	0.00851	yes

G0000002 3067.8		090978-2910072 2						
ENSMUS G0000002 4503.2	Spink3	chr18:43 728068-4373723 7	0	2.83716	100	5.00E-05	0.0047	yes
ENSMUS G0000007 5015.3	Gm1080 1	chr2:986 62236-9866408 3	0	6.24728	100	5.00E-05	0.0047	yes

Supplementary Table S3 - Primer sequences used in the qRT-PCR

S No.	Primer	Sequence
**Human**		
1.	H19	FP: 5’-ACTCAGGAATCGGCTCTGGAA-3’<br>RP: 5’-CTGCTGTTCCGATGGTGTCTT-3’
2.	PCK1	FP: 5’-GGTTCCCAGGGTGCATGAAA-3’<br>RP: 5’-CACGTAGGGTGAATCCGTCAG-3’
3.	G6PC	FP: 5’-TCTACGTCCTCTTCCCCATC-3’<br>RP: 5’-TCAGTATCCAAAACCCACCAG-3’
4.	FBP	FP: 5’-TTTCTGTACCCCGCTAACAAG-3’<br>RP: 5’-TGAATGTCTGTGGGAATGACG-3’
5.	PC	FP: 5’-GACTCTGTGAAACTCGCTAAAC-3’<br>RP: 5’-CTCTGTGACCGTGTGCTC-3’
6.	18S rRNA	FP: 5’-AGAAACGGCTACCACATCCA-3’<br>RP: 5’-CCCTCCAATGGATCCTCGTT-3’
**Mouse**		
1.	H19	FP: 5’-CCTCAAGATGAAAGAAATGGTGCTA-3’<br>RP: 5’-TCAGAACGAGACGGACTTAAAGAA-3’
2.	Igf2	FP: 5’-GCTTGTTGACACGCTTCAGTTTG-3’<br>RP: 5’-GTTGGCACGGCTTGAAGGC-3’
3.	miR-675	RT primer: 5’-CTCAACTGGTGTCGTGGAGTCGGC AATTCAGTTGAGACTGTGGG-3’<br>FP: 5’-ACACTCCAGCTGGGTGGTGCGGAAAGGGCC-3’<br>RP: 5’- GTGTCGTGGAGTCGGCAATTC-3'
4.	β-actin	FP: 5’-AGTGTGACGTTGACATCCGTA-3’<br>RP: 5’-GCCAGAGCAGTAATCTCCTTCT-3’
5.	U6	FP: 5' -GCTTCGGCAGCACATATACTA-3'<br>RP: 5' -AAATATGGAACGCTTCACGA-3'

6.	Pck1	FP: 5' -CCATCCCAACTCGAGATTCTG-3'<br>RP: 5' -CTGAGGGCTTCATAGACAAGG-3'
7.	Fbp1	FP: 5' -CTGATATTCACCGCACTCTGG-3'<br>RP: 5' -CGGCCTTCTCCATGACATAAG-3'
8.	Pcx	FP: 5' -GGACTCCTTTGGACACAGAG-3'<br>RP: 5' -AATCTCATTCTCATACACGTCGG-3'
FP, Forward Primer; RP, Reverse Primer		

Supplementary Figure S1

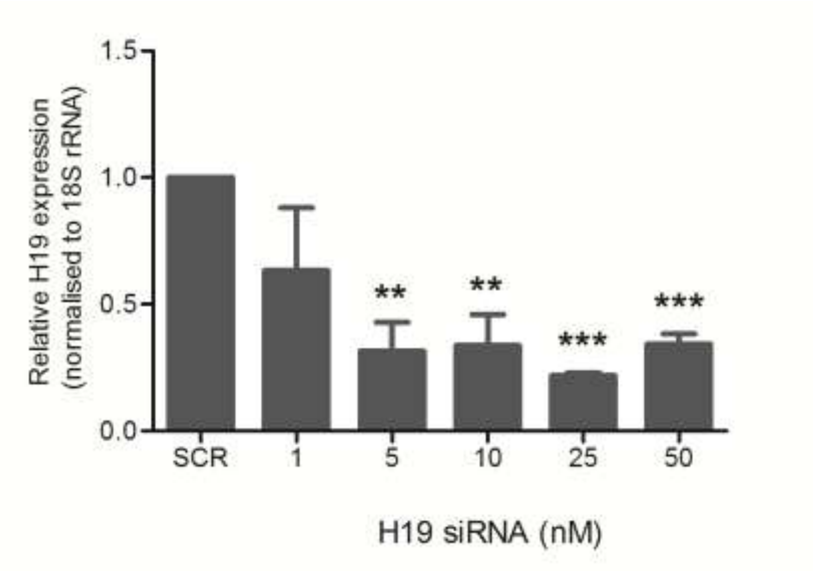

Supplementary Figure S1 - HepG2 cells were reverse transfected with the H19 siRNA (1-50nM) and control cells were transfected with the scramble (SCR), and after 48h of incubation RNA was isolated and assessed for the transcript levels of H19 by qRT-PCR. 18S rRNA was used as a normalization control. Data is presented as mean ± SEM of atleast three independent experiments. **P < 0.01, ***P < 0.001 as compared to scramble transfected cells (SCR).

Supplementary Figure S2 – Full-length blots used to generate Fig. 5c

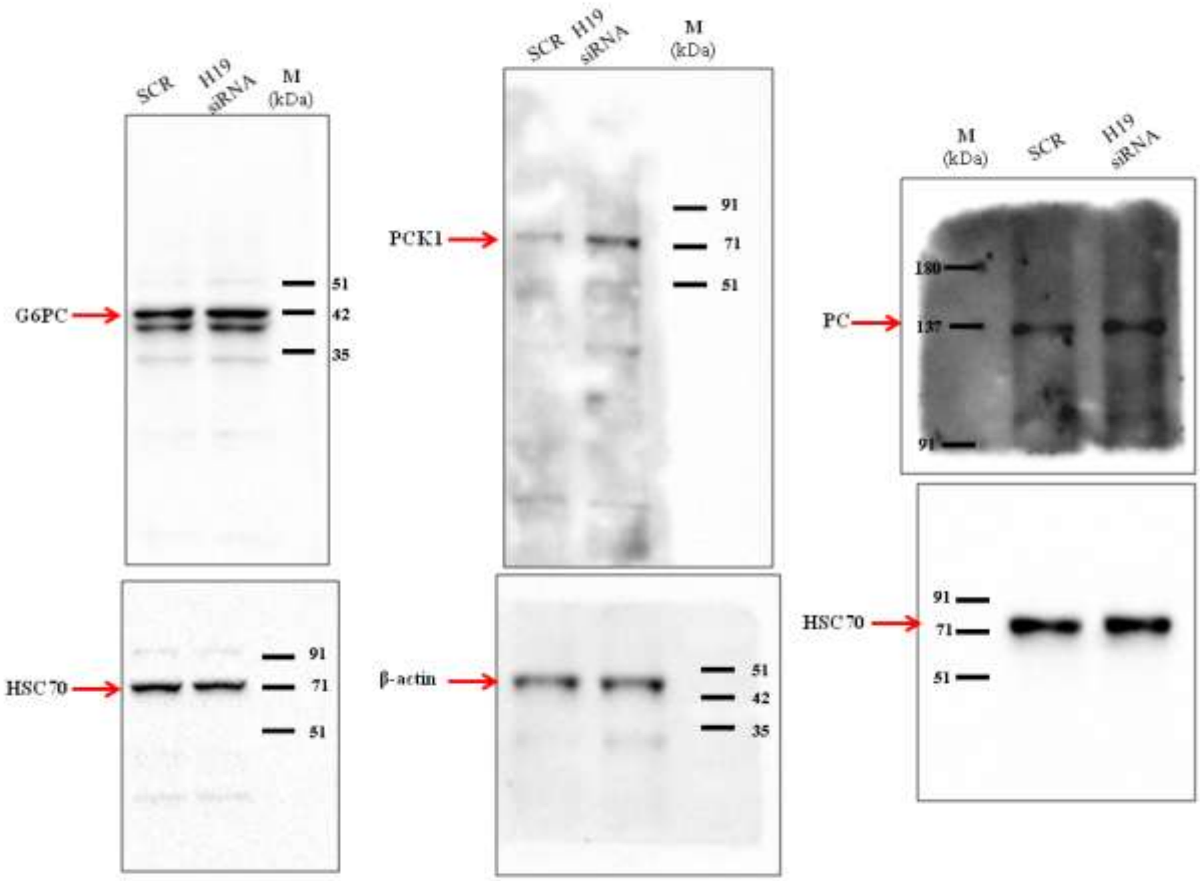

Supplementary Figure S3 – Full-length blots used to generate Fig. 6a

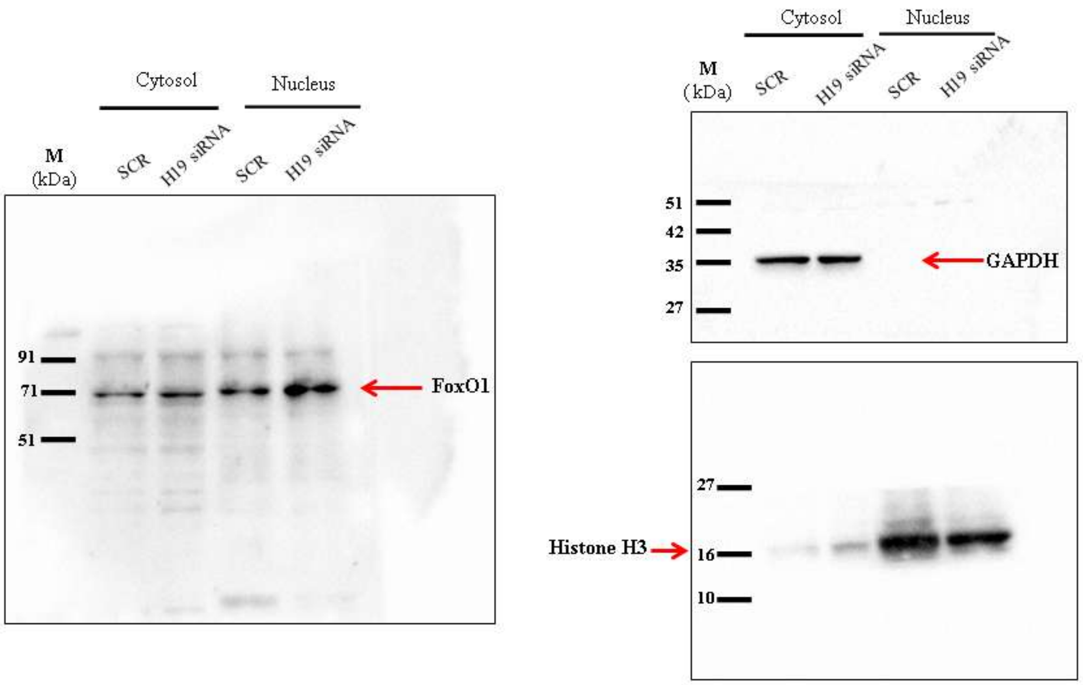

Supplementary Figure S4 – Full-length blots used to generate Fig. 6c

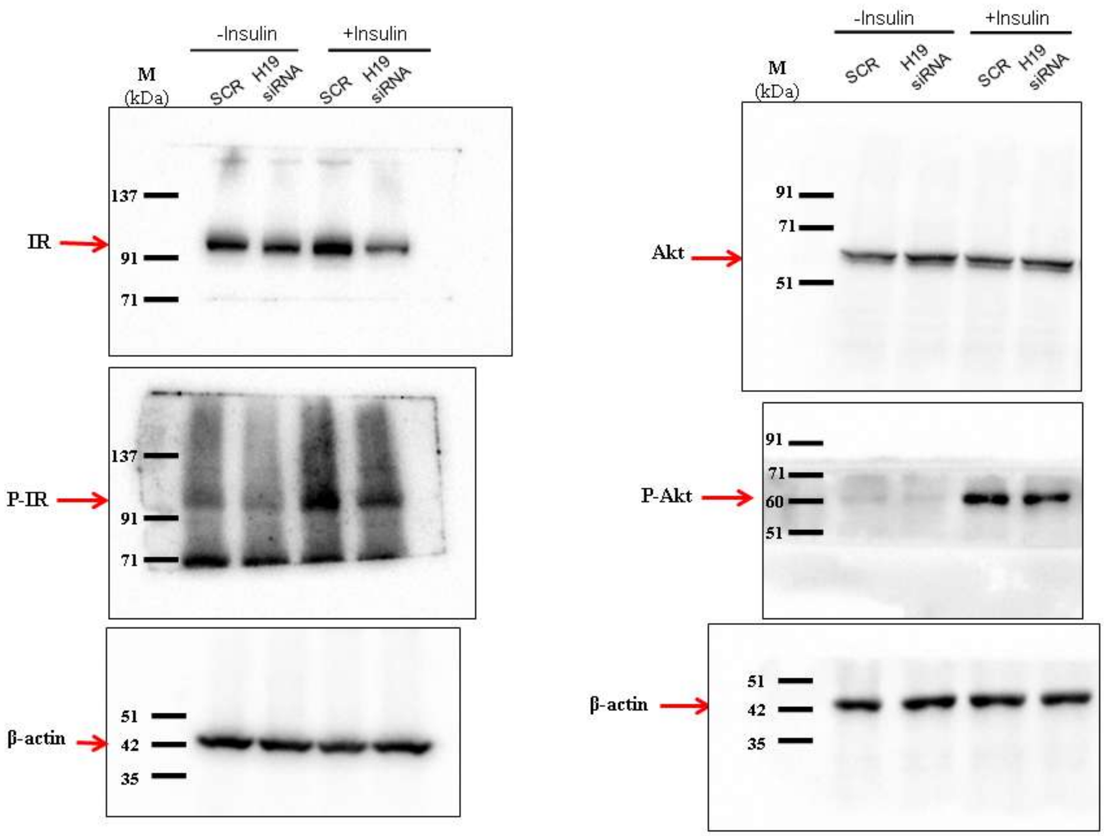

Supplement: Supplementary file 1 — Supplementary information [file 41598_2017_8281_MOESM1_ESM.pdf]
